# Persistent Short Sleep Duration From Pregnancy to 2 to 7 Years After Delivery and Metabolic Health

**DOI:** 10.1001/jamanetworkopen.2024.52204

**Published:** 2024-12-26

**Authors:** Minjee Kim, Laura Elizabeth Wiener, Jace Gilbert, Rebecca B. McNeil, Kathryn J. Reid, William A. Grobman, Francesca Facco, David M. Haas, Robert M. Silver, Philip Greenland, Lynn M. Yee, Phyllis C. Zee

**Affiliations:** 1 Department of Neurology, Feinberg School of Medicine, Northwestern University, Chicago, Illinois; 2 Center for Circadian and Sleep Medicine, Feinberg School of Medicine, Northwestern University, Chicago, Illinois; 3 Center for Applied Health Research on Aging, Institute for Public Health and Medicine, Feinberg School of Medicine, Northwestern University, Chicago, Illinois; 4 Research Triangle Institute International, Research Triangle Park, North Carolina; 5 Department of Obstetrics and Gynecology, The Ohio State College of Medicine, Columbus, Ohio; 6 Department of Obstetrics and Gynecology, University of Pittsburgh, Pittsburgh, Pennsylvania; 7 Department of Obstetrics and Gynecology, Indiana University School of Medicine, Indianapolis; 8 Department of Obstetrics and Gynecology, University of Utah Health Sciences Center, Salt Lake City; 9 Department of Preventive Medicine, Feinberg School of Medicine, Northwestern University, Chicago, Illinois; 10 Department of Obstetrics and Gynecology, Feinberg School of Medicine, Northwestern University, Chicago, Illinois

## Abstract

**Importance:**

Short sleep duration during pregnancy and the perimenopausal period has been associated with adverse cardiometabolic outcomes. However, it remains unclear how sleep duration changes after delivery and whether such changes are associated with the cardiometabolic health of birthing people.

**Objective:**

To investigate whether persistently short sleep during pregnancy and after delivery is associated with incident hypertension and metabolic syndrome.

**Design, Setting, and Participants:**

This secondary analysis of the Nulliparous Pregnancy Outcomes Study: Monitoring Mothers-to-Be Heart Health Study (NuMoM2b-HHS), an ongoing prospective cohort study, was conducted between September 5, 2023, and March 1, 2024, in 8 US academic medical centers. Participants were aged 18 years or older at NuMoM2b enrollment; recruited during their first pregnancy between October 1, 2010, and September 30, 2013; and followed up for a mean (SD) of 3.1 (0.9) years after delivery.

**Exposures:**

Self-reported short sleep duration (<7 hours) during pregnancy and 2 to 7 years after delivery was defined as persistent short sleep.

**Main Outcomes and Measures:**

Incident hypertension and metabolic syndrome (MetS) at follow-up. Regression models were used to estimate relative risks of incident hypertension and MetS by sleep duration pattern. Hypertension analyses excluded participants with hypertension at baseline, and MetS analyses excluded participants with MetS at baseline. Multivariable models included a priori covariates of baseline age and time from delivery to follow-up. Incident hypertension analyses included an additional covariate of body mass index at baseline.

**Results:**

Among 3922 participants (mean [SD] age, 27.3 [5.4] years; 598 Hispanic [15.2%], 485 non-Hispanic Black [12.4%], and 2542 non-Hispanic White [64.8%]), 565 individuals (14.4%) experienced persistent short sleep. Non-Hispanic Black (adjusted odds ratio [aOR], 2.17; 95% CI, 1.59-2.97) and unmarried (aOR, 1.68, 95% CI, 1.29-2.19) participants were significantly more likely to experience persistent short sleep compared with non-Hispanic White and married participants, respectively. Persistent short sleep was associated with higher odds of incident MetS (aOR, 1.60; 95% CI, 1.21-2.11) but not incident hypertension (aOR, 0.91; 95% CI, 0.69-1.19).

**Conclusions and Relevance:**

In this study, short sleep duration that persisted from pregnancy to 2 to 7 years after delivery was associated with a greater risk for adverse cardiometabolic outcomes. Future studies should explore whether sleep-targeted interventions during and after pregnancy are associated with improved cardiometabolic health outcomes, particularly among populations at increased risk.

## Key Points

**Question:**

Is persistently short sleep during pregnancy to 2 to 7 years after delivery associated with the development of incident hypertension or metabolic syndrome in nulliparous pregnant individuals?

**Findings:**

In this cohort study of 3922 nulliparous pregnant people examined during pregnancy and 2 to 7 years after delivery, persistently short sleep duration (<7 hours) was associated with an increased risk of incident metabolic syndrome. There was no association with incident hypertension.

**Meaning:**

This finding suggests that persistently short sleep during and after pregnancy may be an important factor associated with the development of cardiometabolic diseases.

## Teaser

This cohort study investigates the association of persistent short sleep from pregnancy to 2 to 7 years after delivery with hypertension and metabolic syndrome.

## Introduction

Metabolic syndrome (MetS), characterized by the co-occurrence of hypertension (HTN), insulin resistance, obesity, and dyslipidemia, is a key factor associated with cardiovascular morbidity and mortality.^1,2^ The prevalence of MetS has been increasing in the US, especially in women aged 20 to 39 years.^3^ Therefore, there is a need to identify and target modifiable risk factors associated with MetS in this population to reduce the burden of cardiovascular disease,^4^ the leading cause of mortality in US women.^5^

An emerging body of evidence supports associations between short sleep duration and MetS.^6^ Furthermore, major life events unique to women, such as pregnancy and menopause, can affect sleep duration and the risk of MetS.^4^ Short sleep duration during pregnancy is associated with hyperglycemia and gestational diabetes,^7,8,9^ while persistent short sleep across midlife in women who are perimenopausal is associated with incident cardiovascular disease.^10^ However, significant knowledge gaps exist on how sleep duration changes after pregnancy and whether short sleep that persists beyond pregnancy is a risk factor associated with adverse cardiometabolic health. Due to structural factors, historically marginalized groups and those with fewer resources may be at especially high risk of inadequate sleep duration in the years after childbirth, which may influence their long-term health patterns.

Thus, the objectives of this study were to characterize patterns of self-reported sleep duration from pregnancy to 2 to 7 years after delivery and investigate their association with incident HTN and MetS. We hypothesized that non-Hispanic Black race and ethnicity, single marital status, and lower socioeconomic status would be associated with short sleep duration that persisted from pregnancy to the years after delivery and that persistent short sleep duration would be associated with an increased risk of incident HTN and MetS.

## Methods

This is a secondary analysis of data from 2 cohort studies: the Nulliparous Pregnancy Outcomes Study conducted between September 5, 2023, and March 1, 2024: Monitoring Mothers-to-Be (NuMoM2b) and the ongoing NuMoM2b-Heart Health Study (NuMoM2b-HHS). Full study details of NuMoM2b have been described.^11^ Briefly, 10 038 nulliparous individuals (no prior delivery at ≥20 weeks of gestation) in their first trimesters of singleton pregnancy were recruited from 2010 through 2013 at 8 US clinical sites: Case Western Reserve University, Columbia University, Indiana University, the University of Pittsburgh, Northwestern University, the University of California at Irvine, the University of Pennsylvania, and the University of Utah. Study visits 1, 2, and 3 occurred during the following gestational age intervals: 6^0/7^ to 13^6/7^, 16^0/7^ to 21^6/7^, and 22^0/7^ to 29^6/7^ weeks. A subcohort of 3712 NuMoM2b participants underwent evaluation for sleep-disordered breathing (SDB) during the index pregnancy (NuMoM2b SDB substudy).^12,13^ This report followed the Strengthening the Reporting of Observational Studies in Epidemiology (STROBE) reporting guideline for cohort studies. Local institutional review boards approved the study protocol at each clinical site and the Data Coordinating Center, and all participants provided written informed consent. Analyses use data from NuMoM2b and NuMoM2b-HHS. Both NuMoM2b and NuMoM2b-HHS protocols and consents allow for secondary analyses and the publication of these analyses.

### Study Design

Between 2014 and 2017, a total of 4509 participants from NuMoM2b were invited for an in-person visit 2 to 7 years after the index pregnancy ended (visit 5) as part of the NuMoM2b-HHS. Complete methods of NuMoM2b-HHS have been described.^14^ Briefly, participants were asked to fast for 8 hours before the visit. Biological specimens and anthropometric measurements were collected using standardized protocols.^15^ Blood pressure (BP) was measured 3 times, with a 30-second rest period between measurements. Participants were asked to raise their arms with their hands unclenched for at least 5 seconds during each rest period. All 3 measurements were recorded. For analyses, the mean of the last 2 systolic and diastolic pressures was used when 3 measurements were taken. If only 2 values were available, the second value was used. If only 1 value was available, that value was used.^14^

**Figure 1.**
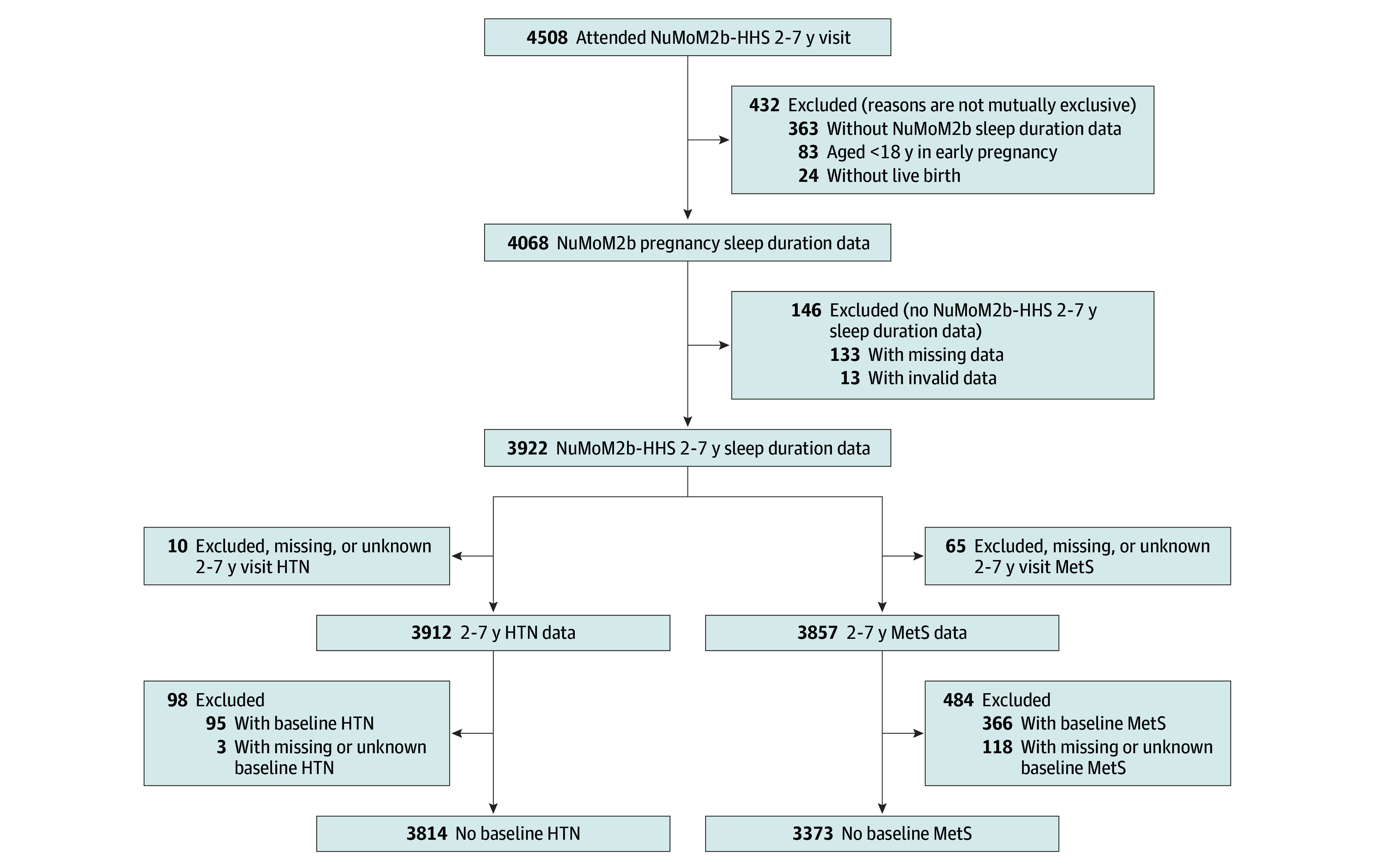
Study Flowchart Enrollment and inclusion of participants in the study are summarized in the flowchart. HTN indicates hypertension; MetS, metabolic syndrome; NuMoM2b, Nulliparous Pregnancy Outcomes Study: Monitoring Mothers-to-Be; nuMoM2b-HHS, nuMoM2b-Heart Health Study.

This analysis was restricted to individuals aged 18 years or older at NuMoM2b enrollment who completed sleep questionnaires at least once during pregnancy (visit 1 or 3) and at follow-up (visit 5) and attended the in-person NuMoM2b-HHS cardiovascular assessment (visit 5) at follow-up. We included only individuals aged 18 years or older because sleep characteristics are significantly different between children and adolescents (ages <18 years) and adults, and the number of participants younger than 18 years was not sufficient to meaningfully adjust for this difference in analysis.^16,17,18^ We excluded individuals who experienced pregnancy loss (spontaneous abortion, induced abortion, or stillbirth) (Figure 1).

### Outcomes

Incident HTN was defined as HTN that developed after the index pregnancy, as determined by BP measurement (systolic BP [SBP] ≥130 mm Hg or diastolic BP [DBP]≥80 mm Hg) or use of an antihypertensive medication for BP control at follow-up. MetS was defined as the presence of 3 or more of the following 5 criteria: waist circumference 80 cm or more (among Asian participants) or 88 cm or more (among other participants); triglyceride levels of 150 mg/dL or more or use of a triglyceride-lowering medication (to convert triglycerides to millimoles per liter, multiply by 0.0113); fasting glucose levels of 100 md/dL or more or use of a glucose-lowering medication (to convert glucose to millimoles per liter, multiply by 0.0555); SBP of 130 mm Hg or more, DBP of 85 mm Hg or more, or use of antihypertensive medication; and high-density lipoprotein levels of less than 50mg/dL or use of high-density lipoprotein–raising medication (to convert high-density lipoprotein to millimoles per liter, multiply by 0.0259).^19^

### Exposure: Sleep Duration Patterns

At study visits 1, 3, and 5, participants were asked about the timing of their sleep on weekdays or workdays and on weekends using the following 2 questions: “Not including naps, what time do you usually go to bed?” and “Not including naps, what time do you usually wake up?” In addition, participants were asked to estimate how many minutes it usually took for them to fall asleep at bedtime and how many minutes of wake time they typically had during a night’s sleep. Calculated sleep duration was estimated as the interval from bedtime to wake time minus the time it took to fall asleep and the time awake during the night. This was separately calculated for weekdays or workdays and for weekends. Quality checks were done on the calculated sleep duration values for weekdays or workdays and for weekends using the following question from the sleep questionnaire: “How many hours of sleep do you usually get per night?” If the reported sleep duration from this additional question was not within 2 hours of the calculated sleep duration, then times used for the calculated sleep duration were run through a series of edit checks to identify and correct potential errors in the selection of am or pm. If the calculated duration was still not within 2 hours of the reported duration after editing potential am or pm errors and recalculating sleep duration, the calculated duration and corresponding times were set to missing. Weekly mean sleep duration was calculated as a weighted mean of weekday or workday and weekend sleep durations using the following formula: ([weekday/workday duration × 5] + [weekend duration × 2])/7).^20^ Based on previous studies demonstrating the association of sleep duration in nonpregnant and pregnant individuals with health outcomes, we selected a cutoff of less than 7 hours a priori to define short sleep duration for our primary analyses.^20,21^ Using this cutoff, we categorized participants by 4 distinct patterns of sleep duration:

- Never short sleep (reference group): sleep duration of 7 hours or more during pregnancy and at the 2- to 7-year visit after delivery (visit 5)
- Persistent short sleep: sleep duration of less than 7 hours during pregnancy and at the 2- to 7-year visit after delivery (visit 5)
- Resolved short sleep: sleep duration of less than 7 hours during pregnancy and 7 or more hours at the 2- to 7-year visit after delivery (visit 5)
- New short sleep: sleep duration of 7 or more hours during pregnancy and less than 7 hours at the 2- to 7-year visit after delivery (visit 5)

Sleep duration during pregnancy was categorized using data from all available visits (ie, visit 1, visit 3, or both). Short sleep duration was defined as reporting less than 7 hours of sleep at least once during the index pregnancy.

### Statistical Analysis

Baseline characteristics of the index pregnancy and cardiovascular characteristics at the follow-up visit were summarized according to sleep duration patterns. We used the χ^2^ or Kruskal-Wallis test for univariate analyses to compare the distribution of sleep duration patterns for categories of self-reported race and ethnicity (as socially constructed variables reflecting a constellation of exposures related to structural racism), marital status, education, and insurance (as a measure of socioeconomic status) at baseline. Participants were asked if they were of Hispanic or Latino origin or descent and which of the following groups described their racial background (participants could answer yes to more than 1 option): Black (African American or African Descent), American Indian or Alaska Native, Asian Indian, Native Hawaiian or Other Pacific Islander, other Asian, White, and other. Based on responses to these questions, 5 groups were constructed: Hispanic, non-Hispanic Asian, non-Hispanic Black, non-Hispanic White, and other (including American Indian or Alaska Native and Native Hawaiian or Other Pacific Islander). Variables associated with sleep duration patterns were further examined in multivariable regression models, adjusting for baseline age and years from delivery of the index pregnancy to the HHS follow-up visit.

Next, we examined associations of sleep duration patterns with incident HTN and MetS using logistic regression. Participants with baseline HTN (ie, SBP ≥130 mm Hg, DBP ≥80 mm Hg, or use of antihypertensive medication for BP control) were excluded from HTN analyses; similarly, individuals who met criteria for MetS at baseline were excluded from MetS analyses. Adjustment covariates, chosen a priori, included years from delivery of the index pregnancy to the HHS follow-up visit, age, and body mass index (calculated as weight in kilograms divided by height in meters squared) in early pregnancy based on their known associations with short sleep duration, HTN, and MetS.^22,23,24,25,26^ MetS analyses did not include body mass index given its causal relationship with increased waist circumference, a component of MetS.

Finally, we conducted a series of sensitivity analyses. First, we tested for interactions between sociodemographic variables and sleep duration patterns in the association with incident HTN and MetS. Second, we examined interactions between sleep duration patterns and antenatal SDB, defined as an apnea-hypopnea index of 5 or greater or an oxygen desaturation index of 5 or greater,^13^ concerning incident HTN and MetS. This analysis included only participants who underwent home sleep apnea tests as part of the NuMoM2b-SDB substudy. The goal was to determine whether any observed associations between sleep duration patterns and these outcomes were mediated by SDB given that SDB can lead to short sleep duration and has been associated with an increased risk of incident MetS after delivery.^13^ Third, we used an alternative cutoff of less than 6 hours to define short sleep.^27^ Lastly, we excluded participants who reported shift work (ie, afternoon, split, night, irregular or on-call, or rotating shift) during the index pregnancy to test whether it was the circadian changes related to shift work rather than short sleep durations that were contributing to any observed associations.^28^

Across analyses, 2-sided, single–degree-of-freedom tests were used at a nominal significance level of α = .05. No correction was made for multiple comparisons. Analyses were conducted using SAS statistical software version 9.4 (SAS Institute).

## Results

Among 3922 birthing people included in the analyses (mean [SD] age at baseline, 27.3 [5.4] years; 598 Hispanic [15.2%], 485 non-Hispanic Black [12.4%], and 2542 non-Hispanic White [64.8%]), 2522 individuals (64.3%) were married at baseline (Table 1). During the index pregnancy, 1013 participants (25.8%) reported short sleep, which increased to 1497 participants (38.2%) at a mean (SD) follow-up of 3.1 (0.9) years, including 565 individuals who had previously reported short sleep in pregnancy (persistent short sleep) and 932 individuals who newly developed short sleep at follow-up (new short sleep). In total, we found persistent short sleep in 565 participants (14.4%), new short sleep in 932 participants (23.8%), resolved short sleep in 448 participants (11.4%), and never short sleep in 1977 participants (50.4%). Overall, the median (IQR) sleep duration decreased from 8.0 (7.3-8.6) hours during the index pregnancy to 7.0 (6.5-8.0) hours at follow-up.

**Table 1.** Sample Characteristics at Baseline

Characteristic	Participants, No. (%)				
	All participants (N = 3922)	Never SS (n = 1977 [50.4%])	Persistent SS (n = 565 [14.4%])	Resolved SS (n = 448 [11.4%])	New SS (n = 932 [23.8%])
Age, y					
Mean (SD)	27.3 (5.4)	27.3 (5.3)	28.0 (5.6)	26.8 (5.6)	27.4 (5.4)
<25	1314 (33.5)	666 (33.7)	172 (30.4)	166 (37.1)	310 (33.3)
25 to 34	2214 (56.5)	1118 (56.6)	318 (56.2)	237 (52.9)	541 (58.0)
>35	392 (10.0)	193 (9.8)	75 (13.3)	45 (10.0)	81 (8.7)
BMI					
Mean (SD)	26.5 (6.4)	25.8 (5.8)	28.0 (7.5)	27.0 (6.6)	27.0 (6.6)
<25	2016 (52.1)	1114 (57.1)	246 (44.0)	212 (48.3)	444 (48.2)
25 to <30	954 (24.7)	464 (23.8)	142 (25.4)	109 (24.8)	239 (26.0)
≥30	900 (23.3)	373 (19.1)	171 (30.6)	118 (26.9)	238 (25.8)
Race and ethnicity					
Asian	122 (3.1)	61 (3.1)	18 (3.2)	7 (1.6)	36 (3.9)
Black non-Hispanic	485 (12.4)	181 (9.2)	108 (19.1)	69 (15.4)	127 (13.6)
Hispanic	598 (15.2)	280 (14.2)	79 (14.0)	85 (19.0)	154 (16.5)
White non-Hispanic	2542 (64.8)	1369 (69.2)	334 (59.1)	265 (59.2)	574 (61.6)
Other^a^	175 (4.5)	86 (4.4)	26 (4.6)	22 (4.9)	41 (4.4)
Education					
<High school graduate	185 (4.7)	92 (4.7)	27 (4.8)	27 (6.0)	39 (4.2)
High school graduate or GED	431 (11.0)	206 (10.4)	69 (12.2)	64 (14.3)	92 (9.9)
Some college credit, no degree	779 (19.9)	360 (18.2)	130 (23.0)	103 (23.0)	186 (20.0)
Associate or technical degree	454 (11.6)	194 (9.8)	85 (15.0)	62 (13.8)	113 (12.1)
Bachelor’s degree	1169 (29.8)	632 (32.0)	135 (23.9)	110 (24.6)	292 (31.3)
Degree beyond bachelor’s	904 (23.0)	493 (24.9)	119 (21.1)	82 (18.3)	210 (22.5)
Marital status					
Married	2522 (64.3)	1359 (68.7)	311 (55.0)	239 (53.3)	613 (65.8)
Single or never married	1363 (34.8)	606 (30.7)	240 (42.5)	205 (45.8)	312 (33.5)
Separated, divorced, or widowed	37 (0.9)	12 (0.6)	14 (2.5)	4 (0.9)	7 (0.8)
Commercial insurance	2771 (71.0)	1434 (72.8)	404 (72.1)	280 (62.9)	653 (70.3)
Employed in early pregnancy	2802 (82.4)	1427 (83.6)	407 (84.3)	318 (78.7)	650 (80.6)
Shift work	737 (26.3)	353 (24.7)	137 (33.7)	95 (29.8)	152 (23.4)
Employed in mid-pregnancy	2990 (79.8)	1531 (80.8)	438 (80.4)	333 (77.8)	688 (78.0)
Shift work	762 (28.1)	345 (25.3)	148 (37.7)	94 (31.1)	175 (27.0)
Self-report sleep duration, median (IQR), h					
Early pregnancy	8.0 (7.3-8.6)	8.3 (7.9-9.0)	6.6 (6.0-7.3)	6.9 (6.3-8.0)	8.0 (7.6-8.6)
Midpregnancy	8.0 (7.0-8.4)	8.0 (7.6-8.7)	6.3 (5.9-6.7)	6.6 (6.0-7.0)	8.0 (7.3-8.4)
2 to 7 y After delivery	7.0 (6.5-8.0)	7.6 (7.3-8.0)	6.0 (5.5-6.5)	7.5 (7.0-8.0)	6.3 (6.0-6.6)

Abbreviations: BMI, body mass index (calculated as weight in kilograms divided by height in meters squared); GED, general educational development certificate; SS, short sleep.

^a^ Other includes American Indian or Alaska Native and Native Hawaiian or Other Pacific Islander.

### Sociodemographic Variables Associated With Sleep Duration Patterns

**Figure 2.**
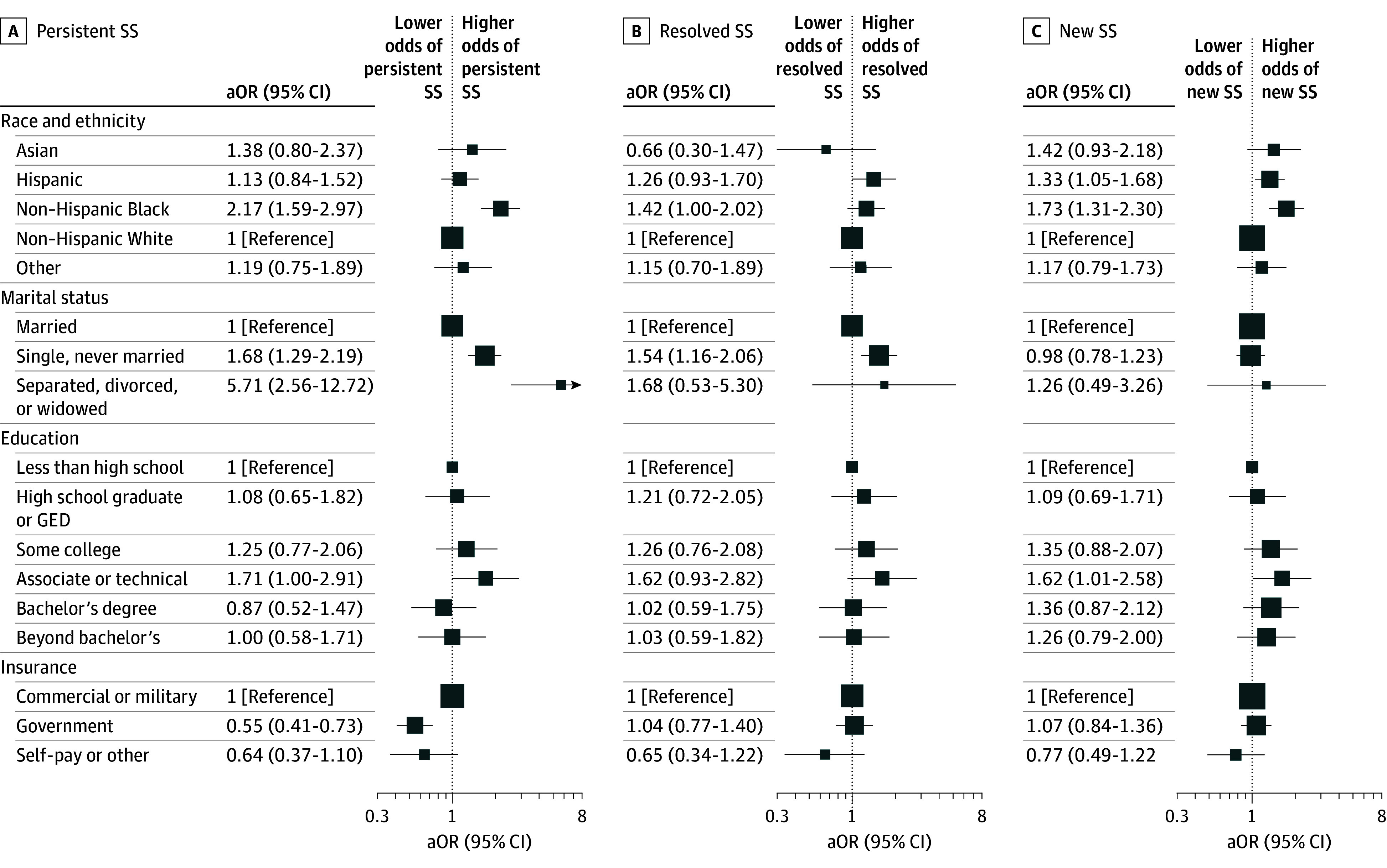
Association of Sociodemographic Characteristics With Short Sleep (SS) Duration Patterns Associations between early pregnancy sociodemographic characteristics in the Nulliparous Pregnancy Outcomes Study: Monitoring Mothers-to-Be and SS duration patterns are summarized as adjusted odds ratios (aORs) with 95% CIs after adjustment for baseline age and time from delivery to follow-up study visit. GED indicates general education development certificate.

Compared with non-Hispanic White participants, non-Hispanic Black individuals were more likely to report persistent (adjusted odds ratio [aOR], 2.17; 95% CI, 1.59-2.97) or new (aOR, 1.73; 95% CI, 1.31-2.30) short sleep. Hispanic participants were more likely to develop new short sleep (aOR, 1.33; 95% CI, 1.05-1.68) compared with non-Hispanic White participants. Put another way, 235 non-Hispanic Black (48.5%) and 233 Hispanic (39.0%) participants reported short sleep at follow-up compared with 908 non-Hispanic White individuals (35.7%). In addition, single, never-married (aOR, 1.68; 95% CI, 1.29-2.19) and separated, divorced, or widowed (aOR, 5.71; 95% CI, 2.56-12.72) participants were more likely to report persistent short sleep than married participants. Sleep duration patterns were not associated with educational attainment or insurance status (Figure 2; eTable 1 in Supplement 1).

### Cardiometabolic Outcomes and Associations With Sleep Duration Patterns

**Figure 3.**
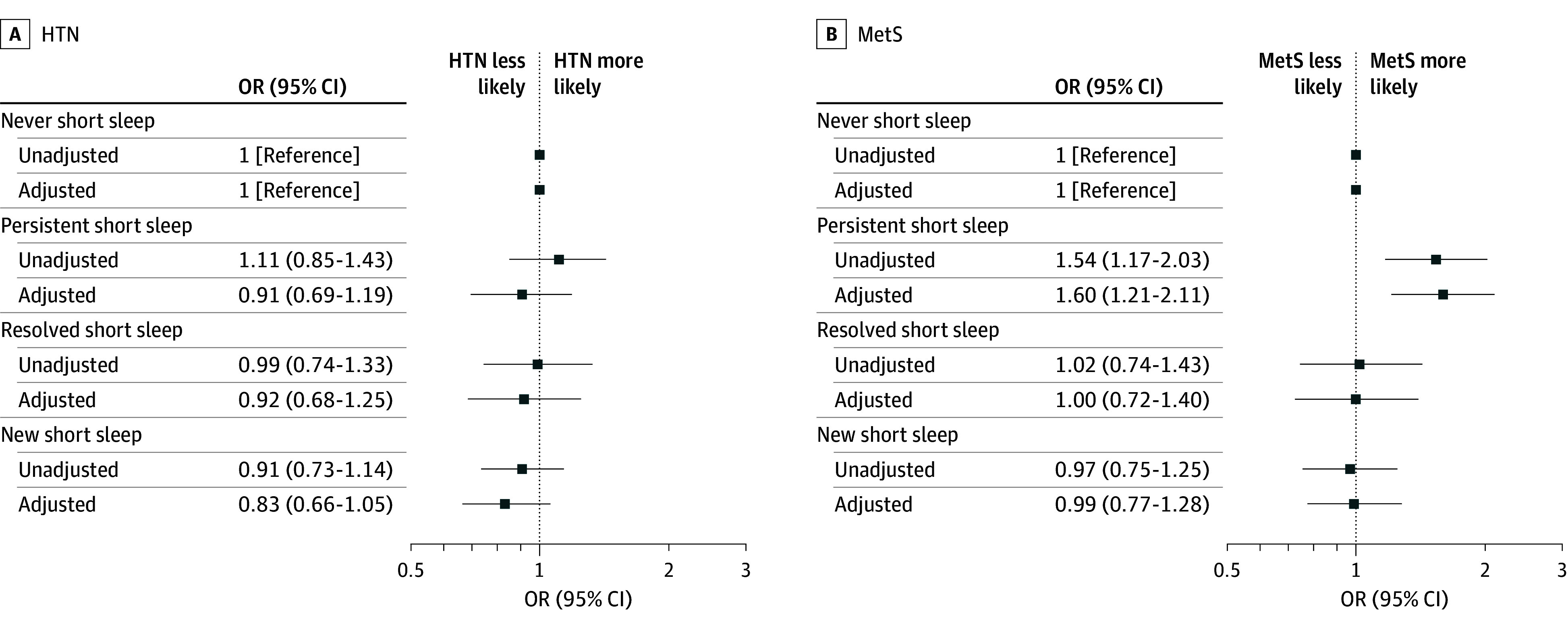
Association of Sleep Duration Patterns With Incident Hypertension (HTB) and Metabolic Syndrome (MetS) Associations of sleep duration patterns (reference group: never short sleep) with incident HTN (A) or MetS (B) 2 to 7 years after index pregnancy are summarized as unadjusted and adjusted odds ratios (ORs) with 95% CIs. Adjustment covariates included age and body mass index (calculated as weight in kilograms divided by height in meters squared) in early index pregnancy and time from delivery to follow-up study visit for HTN (A) and age in early index pregnancy and time from delivery to follow-up study visit for MetS (B).

Baseline cardiometabolic measures are summarized in eTable 2 in Supplement 1. Among 3336 participants without baseline HTN, 599 participants (18.0%) met criteria for HTN at follow-up (Table 2). Sleep duration patterns were not associated with incident HTN in univariable or multivariable analyses (eg, persistent short sleep: OR, 1.11; 95% CI, 0.85-1.43 and aOR, 0.91; 95% CI, 0.69-1.19, respectively) (Figure 3; eTable 3 in Supplement 1). Among 3373 participants without baseline MetS, 447 participants (13.3%) developed MetS at follow-up (Table 2). Persistent short sleep was associated with incident MetS (unadjusted OR, 1.54; 95% CI, 1.17-2.03), which remained significant after adjusting for baseline age and time from delivery to follow-up (aOR, 1.60; 95% CI, 1.21-2.11) (Figure 3; eTable 3 in Supplement 1).

**Table 2.** Cardiovascular Characteristics 2-7 y After Index Pregnancy

Characteristic	Participants, No. (%)				
	Overall (N = 3922)	Never SS (n = 1977 [50.4%])	Persistent SS (n = 565 [14.4%])	Resolved SS (n = 448 [11.4%])	New SS (n = 932 [23.8%])
BMI					
Participants with data, No.	3900	1966	562	444	928
Mean (SD)	27.7 (7.6)	26.8 (7.1)	29.5 (8.5)	28.3 (7.7)	28.1 (7.7)
<25	1801 (46.2)	1003 (51.0)	211 (37.5)	182 (41.0)	405 (43.6)
25 to 30	948 (24.3)	467 (23.8)	128 (22.8)	111 (25.0)	242 (26.1)
≥30	1151 (29.5)	496 (25.2)	223 (39.7)	151 (34.0)	281 (30.3)
Waist circumference					
Participants with data, No.	3904	1972	562	445	925
Mean (SD), cm	96.0 (16.3)	94.5 (15.0)	99.1 (18.3)	96.8 (17.3)	97.0 (16.5)
≥80 cm (Asian participants) or ≥88 cm (other participants)	2525 (64.7)	1224 (62.1)	393 (69.9)	286 (64.3)	622 (67.2)
Blood pressure					
Participants with data, No.	3912	1973	563	448	928
SBP, mean (SD), mm Hg	111.4 (10.9)	111.2 (11.0)	112.2 (10.8)	111.6 (10.8)	111.1 (11.0)
DBP, mean (SD), mm Hg	72.0 (9.9)	72.0 (10.0)	73.0 (9.7)	72.1 (9.7)	71.5 (9.7)
Prevalent HTN, No./total No. (%)^a^	838/3912 (21.4)	428/1973 (21.7)	136/563 (24.2)	90/448 (20.1)	184/928 (19.8)
Incident HTN, No./total No. (%)^a^	599/3336 (18.0)	309/1710 (18.1)	92/469 (19.6)	66/368 (17.9)	132/789 (16.7)
Triglycerides					
Participants with data, No.	3861	1942	555	442	921
Mean (SD), mg/dL	95.0 (58.0)	95.3 (58.6)	100.3 (60.4)	93.6 (53.3)	92.0 (57.4)
≥150 mg/dL Or taking lipid-lowering medication	470 (12.2)	236 (12.2)	80 (14.4)	51 (11.5)	103 (11.2)
HDL cholesterol	56.1 (13.4)	56.4 (13.4)	55.1 (13.5)	55.6 (12.8)	56.1 (13.4)
Participants with data, No.	3859	1941	554	441	923
Mean (SD), mg/dL	56.1 (13.4)	56.4 (13.4)	55.1 (13.5)	55.6 (12.8)	56.1 (13.4)
<50 mg/dL or taking HDL-raising medication	1297 (33.6)	634 (32.7)	210 (37.9)	150 (34.0)	303 (32.9)
Fasting blood glucose					
Participants with data, No.	3317	1674	479	379	785
Mean (SD), mg/dL	91.0 (21.3)	90.7 (19.4)	93.2 (27.9)	89.2 (12.6)	91.4 (23.8)
≥100 mg/dL or taking glucose-lowering medication	623 (16.2)	293 (15.1)	108 (19.6)	70 (15.9)	152 (16.5)
Prevalent MetS, No./total No. (%)^b^	627/3857 (16.3)	292/1943 (15.0)	126/554 (22.7)	71/441 (16.1)	138/919 (15.0)
Incident MetS, No./total No. (%)^b^	447/3373 (13.3)	214/1709 (12.5)	86/475 (18.1)	49/383 (12.8)	98/806 (12.2)
Years from delivery to HHS cardiovascular assessment, mean (SD)	3.1 (0.9)	3.1 (0.9)	3.2 (0.9)	3.1 (0.9)	3.2 (0.8)

Abbreviations: BMI, body mass index (calculated as weight in kilograms divided by height in meters squared); DBP, diastolic blood pressure; HDL, high-density lipoprotein; HHS, Heart Healthy Study; HTN, hypertension; MetS, metabolic syndrome; SBP, systolic blood pressure; SS, short sleep.

SI conversion factors: To convert glucose to millimoles per liter, multiply by 0.0555; HDL to millimoles per liter, multiply by 0.0259; triglycerides to millimoles per liter, multiply by 0.0113.

^a^ HTN was defined as an SBP of 130 or greater, a DBP of 80 or greater, or the use of an antihypertensive medication. For prevalent HTN, totals (denominators) are the number of participants with data; for incident HTN, totals (denominators) are the number of participants with data excluding those with baseline HTN.

^b^ MetS was defined based on the presence of 3 of 5 of the following criteria: elevated waist circumference, elevated triglyceride levels or associated medication, elevated fasting glucose levels or associated medication, elevated blood pressure (SBP ≥130 or DBP ≥85) or associated medication, and reduced HDL cholesterol levels or associated medication. For prevalent MetS, totals (denominators) are the number of participants with data; for incident MetS, totals (denominators) are the number of participants with data excluding those with baseline MetS.

### Sensitivity Analyses

No interaction was found between sleep duration patterns, race and ethnicity, or marital status concerning incident HTN or MetS. In the subset of 1368 participants who underwent a home sleep apnea test as part of the NuMoM2b-SDB substudy, no interaction existed between sleep duration patterns and SDB concerning incident HTN or MetS, whether SDB was defined by an apnea-hypopnea index of 5 or greater or an oxygen desaturation index of 5 or greater (eTable 4 in Supplement 1).

When we defined short sleep duration using the criteria of less than 6 hours, we found persistent, resolved, and new short sleep in 99 participants (2.5%), 210 participants (5.4%), and 294 participants (7.5%), respectively. Among sociodemographic characteristics, only marital status was associated with persistent short sleep duration of less than 6 hours (eTable 5 in Supplement 1). Compared with never short sleep (<6 hours), persistent (aOR, 2.31; 95% CI, 1.36-3.93), new (aOR, 2.12; 95% CI, 1.55-2.91), and resolved (aOR, 1.73; 95% CI, 1.16-2.57) short sleep were all associated with greater odds of incident MetS after adjusting for age and time from delivery to follow-up (eFigure and eTable 6 in Supplement 1). No association was found between short sleep duration patterns (<6 hours) and incident HTN. Lastly, exclusion of 1010 participants who reported shift work during the index pregnancy did not meaningfully change results (eTable 7 in Supplement 1).

## Discussion

In this cohort study of a geographically and socioeconomically diverse sample of nulliparous birthing people who completed self-reported sleep assessments during their pregnancy and at a mean follow-up of 3.1 years after delivery, 14.4% reported short sleep duration (<7 hours) that persisted from pregnancy to after delivery. Persistent short sleep duration was associated with 1.6-fold higher odds of incident MetS but was not associated with odds of incident HTN. This association was not explained by SDB or shift work. Notably, more extreme short sleep (<6 hours) was associated with an even greater increase in odds of developing MetS. Under this definition, participants with persistent short sleep had 2.3-fold higher odds, while those with new or resolved short sleep had 2.1-fold and 1.7-fold higher odds, respectively, of incident MetS.

Our findings suggest that exposure to self-reported short sleep duration in pregnancy and following years was associated with higher odds of incident MetS in a dose-dependent manner. These findings are consistent with prior reports associating inadequate sleep duration with cardiometabolic dysfunction. In a meta-analysis^6^ including 195 902 participants, self-reported short sleep duration at baseline (mean age, 40.0 years) was associated with 1.3-fold higher odds of incident MetS at a mean follow-up of 3.2 years. Another meta-analysis^29^ including 196 041 participants demonstrated that each 1-hour shorter sleep duration from 7 hours was associated with a 9% higher risk of incident obesity. These associations were modified by age and sex, suggesting stronger associations between short sleep and metabolic dysregulation in younger individuals (aged <60 years) and women.

Among investigations focused on women, the Study of Women’s Health Across the Nation (SWAN) assessed self-reported sleep duration 4 times over 13 years among premenopausal and early perimenopausal women aged 42 to 52 years at baseline.^30^ SWAN found that chronic exposure to short sleep across midlife was associated with a higher risk of incident cardiovascular diseases.^10^ Studies of pregnant people have reported mixed results. A previous study^20^ that analyzed the same NuMoM2b cohort as our study did not observe associations between self-reported short sleep duration and gestational diabetes,^20^ in contrast to other studies of pregnant individuals.^31^ These conflicting results may be partially explained by differences in the study population given that the NuMoM2b cohort was limited to nulliparous individuals, while the risk of gestational diabetes increases with parity, age, and a prior history of gestational diabetes. A few studies have examined cardiometabolic outcomes associated with postpartum sleep duration, largely focused on the first year after delivery. Short sleep duration in this period was associated with higher postpartum weight retention, adiposity, and waist circumference.^32,33^ However, short sleep duration during the first postpartum year may be a time-limited phenomenon in some individuals, especially if explained by infant care and feeding responsibilities.^34,35,36^ By reporting the prevalence and metabolic significance of persistent short sleep in the years after a first birth, our study fills an existing knowledge gap in this life stage.

Several longitudinal studies, including the SWAN cohort,^30,37^ observed the stability of sleep duration across midlife, suggesting that many individuals establish their habitual sleep at an earlier life stage. In our sample, we observed significant changes in sleep duration from pregnancy to after delivery. In addition to an overall decrease in the median sleep duration from 8.0 during pregnancy to 7.0 hours after delivery, we found that more than one-third of our sample exhibited categorical changes in sleep duration, reporting resolved (11.4%) or new-onset (23.8%) short sleep at follow-up. Changes in sleep duration after childbirth have been observed in other cohort studies, including a Norwegian birth cohort^35^ in which the mean sleep duration changed from 7.3 hours in late pregnancy to 6.5 hours at 8 weeks after delivery, then to 6.9 hours at 2 years after delivery. Given that MetS is considered a modifiable risk factor associated with cardiometabolic diseases, our findings highlight the importance of pregnancy and early parenting years as an optimal time window to address short sleep duration. This perspective is supported by a 2022 randomized clinical trial^38^ demonstrating that behavioral interventions, such as personalized sleep hygiene counseling, effectively increased sleep duration among adults with overweight who had habitual short sleep (<6.5 hours). This intervention led to a significant reduction in daily caloric intake without altering energy expenditure, with an inverse correlation between the change in sleep duration and the change in caloric intake. Future studies should consider developing and testing similar sleep extension interventions tailored to the unique challenges people face in pregnancy and early parenthood, particularly in resource-limited settings.

Although we observed an overall reduction in sleep duration from pregnancy to after delivery, exposure to short sleep was unevenly distributed across racial and ethnic groups. Compared with non-Hispanic White individuals, non-Hispanic Black participants were more than twice as likely to experience persistent short sleep and nearly twice as likely to develop new short sleep after delivery. Put another way, 48.5% of non-Hispanic Black participants reported short sleep at follow-up compared with 35.7% of non-Hispanic White individuals. This finding is in line with the growing body of literature demonstrating the disproportionate burden of inadequate sleep duration in historically minoritized groups in the US.^39^ In particular, prior research observed the greatest disparities in sleep duration among non-Hispanic Black women ages 25 to 49 years,^39^ suggesting heightened risk in this group during the most common years for pregnancy and early parenting.

Potential mechanisms may include that non-Hispanic Black mothers are disproportionately affected by structural racism and racial discrimination.^40^ These factors contribute to higher rates of mental health concerns, such as anxiety, depression, and posttraumatic stress disorder, which are further compounded by barriers to mental health care, such as limited access, misdiagnoses, clinician bias, and cultural stigma.^41,42^ Economic disparities may also play a significant role given that non-Hispanic Black mothers are more likely to experience reduced job security and a lack of paid maternity leave, leading to financial instability.^43^ This instability heightens stress related to meeting basic needs, such as housing, food, and childcare.^44,45,46^ Additionally, residential segregation, a consequence of structural racism, often leads to non-Hispanic Black mothers living in neighborhoods exposed to environmental stressors like noise, air pollution, and crime,^47^ which significantly increases parenting stress and contributes to physical inactivity.^48^ These combined factors can negatively impact sleep duration and overall sleep health. Thus, the years after childbirth represent a life stage in which sleep disparities may result in shifts in long-term health patterns. Although data from this study are limited in elucidating potential mechanisms, future research should investigate determinants of sleep disparities in this period, which may inform the development of culturally appropriate interventions.^49^

Inadequate sleep duration has been associated with several physiologic and behavioral mechanisms that may be associated with increased risk of cardiometabolic disorders. Sleep restriction can alter hormonal signaling,^50,51^ which may contribute to increased appetite, altered eating behaviors, and, ultimately, the development of insulin resistance, a core feature of MetS.^29,52^ Additional outcomes associated with chronic sleep insufficiency, such as autonomic dysregulation,^53,54^ inflammation,^55,56^ and endothelial dysfunction,^57^ may further contribute to poor cardiometabolic health. Previous studies demonstrated an association between short sleep duration and incident HTN.^58,59^ This includes the Whitehall II Study,^60^ in which sleep duration of less than 6 hours in women aged 47 to 57 years was associated with 1.4-fold higher odds of incident HTN at a mean follow-up of 5 years. Our null findings, which contrast with prior reports, may be related to a study population that had younger participants and a shorter follow-up interval. Further longitudinal observations are necessary to better elucidate the association between sleep health and cardiometabolic health.

### Limitations

This study has several limitations. First, sleep duration was measured by self-report, which is subject to recall bias and over or underestimation. However, in a study^20^ that examined a subset of the same NuMoM2b cohort as this study, 77.1% of the 752 participants showed concordance between self-reported and objective, actigraphy-measured sleep duration (categorized as <7 or ≥7 hours). Second, we could not establish the temporality between sleep duration patterns and the onset of outcomes given that exposure and outcome measures were collected over the same period. Specifically, we lack data on the cumulative burden of insufficient sleep prior to the onset of HTN or MetS within each sleep duration pattern, although we speculate that this burden was highest in the persistent short sleep group and lowest in the never short sleep group. Third, we did not have comprehensive data on other important sleep characteristics, such as regularity and satisfaction, given that the follow-up used abbreviated sleep questionnaires. This limitation reduced our capacity to fully explore the multifaceted nature of sleep health and its association with cardiometabolic outcomes. Fourth, our ability to understand mechanisms underlying observed associations and to guide targeted interventions was restricted by the absence of several key factors that may be associated with sleep. These include prepregnancy sleep characteristics, duration and methods of infant feeding (formula vs breastfeeding), parenting or childcare support (particularly at night), mental health conditions, use of medications or substances affecting sleep, and time constraints associated with household, work, and other responsibilities.

Fifth, regarding the definition of MetS, we used adjusted waist circumference cutoffs for Asian participants to more accurately reflect cardiovascular risk. This adjustment was based on data showing that Asian populations tend to have greater abdominal adiposity and body fat at lower waist circumference values.^61,62^ Although this method aligns with major guidelines that recommend population-specific thresholds for abdominal obesity,^61^ accounting for well-documented biological differences in body composition across racial and ethnic groups, it may unintentionally reflect biological essentialism. This stands in contrast to our broader view of race and ethnicity as socially constructed variables.

Sixth, in terms of external validity, the study sample included only nulliparous individuals who received care at US academic medical centers and initiated prenatal care in the first trimester, limiting the generalizability of our findings to other birthing populations. Seventh, the relatively short follow-up interval may have contributed to the absence of an association between persistently short sleep and incident HTN. Future longitudinal studies, including the ongoing NuMoM2b-HHS-2 study,^63^ will provide further insight into the complex association between sleep and cardiometabolic health across birthing people’s life courses.

## Conclusions

In this cohort study of nulliparous birthing people, persistent short sleep duration from pregnancy to 2 to 7 years after delivery was associated with an increased risk of developing metabolic syndrome. This underscores the peripartum period as a critical life stage during which inadequate sleep may contribute to metabolic dysregulation and long-term cardiovascular risk. Additionally, racial and ethnic disparities in sleep duration were apparent in this period, which may contribute to lifelong health inequity. These findings highlight the need for targeted interventions aimed at improving sleep health among populations at increased risk to mitigate adverse health outcomes and to promote health equity.

## Notes

Supplement 1. **eTable 1.** Association of NuMoM2b Sociodemographic Characteristics With Short Sleep Duration Patterns **eTable 2.** Biological Measures of Hypertension and Metabolic Risk at First Pregnancy Visit According to Short Sleep Duration Patterns **eTable 3.** Association of Short Sleep Duration Patterns With Incident Hypertension and Metabolic Syndrome 2-7 y After Index Pregnancy **eTable 4.** Association of Sleep-Disordered Breathing and Short Sleep Duration Patterns (Defined as <7 hr) With Incident Hypertension and Metabolic Syndrome 2-7 y After Index Pregnancy **eTable 5.** Association of NuMoM2b Sociodemographic Characteristics With Short Sleep Duration Patterns (Defined as <6 hr) **eFigure.** Association of Short Sleep Duration Patterns (Defined as <6 hr) With Incident Hypertension and Metabolic Syndrome 2-7 y After Index Pregnancy **eTable 6.** Association of Short Sleep Duration Patterns (Defined as <6 hr) With Incident Hypertension and Metabolic Syndrome 2-7 y After Index Pregnancy **eTable 7.** Association of Short Sleep Duration Patterns (Defined as <7 hr) With Incident Hypertension and Metabolic Syndrome 2-7 y After Index Pregnancy Excluding Participants Who Reported Shift Work During Pregnancy

Supplement 2. **Nonauthor Collaborators.** Eunice Kennedy Shriver National Institute of Child Health and Human Development NuMoM2b and National Heart, Lung, and Blood Institute NuMoM2b Heart Health Study Network

Supplement 3.
Data Sharing Statement
