# Supplementary material for: Persistent Short Sleep Duration From Pregnancy to 2 to 7 Years After Delivery and Metabolic Health
Source: JAMA Netw Open. 2024 Dec 26;7(12):e2452204. doi: 10.1001/jamanetworkopen.2024.52204 (PMC11672157; doi:10.1001/jamanetworkopen.2024.52204)
Supplement: Supplement 1. — eTable 1. Association of NuMoM2b Sociodemographic Characteristics With Short Sleep Duration Patterns eTable 2. Biological Measures of Hypertension and Metabolic Risk at First Pregnancy Visit According to Short Sleep Duration Patterns eTable 3. Association of Short Sleep Duration Patterns With Incident Hypertension and Metabolic Syndrome 2-7 y After Index Pregnancy eTable 4. Association of Sleep-Disordered Breathing and Short Sleep Duration Patterns (Defined as <7 hr) With Incident Hypertension and Metabolic Syndrome 2-7 y After Index Pregnancy eTable 5. Association of NuMoM2b Sociodemographic Characteristics With Short Sleep Duration Patterns (Defined as <6 hr) eFigure. Association of Short Sleep Duration Patterns (Defined as <6 hr) With Incident Hypertension and Metabolic Syndrome 2-7 y After Index Pregnancy eTable 6. Association of Short Sleep Duration Patterns (Defined as <6 hr) With Incident Hypertension and Metabolic Syndrome 2-7 y After Index Pregnancy eTable 7. Association of Short Sleep Duration Patterns (Defined as <7 hr) With Incident Hypertension and Metabolic Syndrome 2-7 y After Index Pregnancy Excluding Participants Who Reported Shift Work During Pregnancy [file jamanetwopen-e2452204-s001.pdf]

Supplemental Online Content

Kim M, Wiener LE, Gilbert J, et al; for the Eunice Kennedy Shriver National Institute of Child Health and Human Development NuMoM2b and National Heart, Lung, and Blood Institute NuMoM2b Heart Health Study Network. Persistent short sleep duration from pregnancy to 2 to 7 years after delivery and metabolic health. *JAMA Netw Open*. 2024;7(12):e2452204. doi:10.1001/jamanetworkopen.2024.52204

eTable 1. Association of NuMoM2b Sociodemographic Characteristics With Short Sleep Duration Patterns
eTable 2. Biological Measures of Hypertension and Metabolic Risk at First Pregnancy Visit According to Short Sleep Duration Patterns
eTable 3. Association of Short Sleep Duration Patterns With Incident Hypertension and Metabolic Syndrome 2-7 y After Index Pregnancy
eTable 4. Association of Sleep-Disordered Breathing and Short Sleep Duration Patterns (Defined as <7 hr) With Incident Hypertension and Metabolic Syndrome 2-7 y After Index Pregnancy
eTable 5. Association of NuMoM2b Sociodemographic Characteristics With Short Sleep Duration Patterns (Defined as <6 hr)
eFigure. Association of Short Sleep Duration Patterns (Defined as <6 hr) With Incident Hypertension and Metabolic Syndrome 2-7 y After Index Pregnancy
eTable 6. Association of Short Sleep Duration Patterns (Defined as <6 hr) With Incident Hypertension and Metabolic Syndrome 2-7 y After Index Pregnancy
eTable 7. Association of Short Sleep Duration Patterns (Defined as <7 hr) With Incident Hypertension and Metabolic Syndrome 2-7 y After Index Pregnancy Excluding Participants Who Reported Shift Work During Pregnancy

This supplemental material has been provided by the authors to give readers additional information about their work.

© 2024 Kim M et al. *JAMA Network Open.*

eTable 1. Association of nuMoM2b Sociodemographic Characteristics with Short Sleep Duration Patterns

Baseline Characteristics	Unadjusted Models			Adjusted Models†		
	Persistent vs Never, OR (95% CI)	Resolved vs Never, OR (95% CI)	New vs Never, OR (95% CI)	Persistent vs Never, OR (95% CI)	Resolved vs Never, OR (95% CI)	New vs Never, OR (95% CI)
**Race and ethnicity**						
White non-Hispanic	Ref	Ref	Ref	Ref	Ref	Ref
Black non-Hispanic	**2.45 (1.87, 3.19)**	**1.97 (1.45, 2.68)**	**1.67 (1.31, 2.14)**	**2.17 (1.59, 2.97)**	**1.42 (1.00, 2.02)^**	**1.73 (1.31, 2.30)**
Hispanic	1.16 (0.88, 1.53)	**1.57 (1.19, 2.07)**	**1.31 (1.05, 1.63)**	1.13 (0.84, 1.52)	1.26 (0.93, 1.70)	**1.33 (1.05, 1.68)**
Asian	1.21 (0.71, 2.07)	0.59 (0.27, 1.31)	1.41 (0.92, 2.15)	1.38 (0.80, 2.37)	0.66 (0.30, 1.47)	1.42 (0.93, 2.18)
Other	1.24 (0.79, 1.95)	1.32 (0.81, 2.15)	1.14 (0.77, 1.67)	1.19 (0.75, 1.89)	1.15 (0.70, 1.89)	1.17 (0.79, 1.73)
**Marital status**						
Married	Ref	Ref	Ref	Ref	Ref	Ref
Single, never married	**1.73 (1.43, 2.10)**	**1.92 (1.56, 2.37)**	1.14 (0.97, 1.35)	**1.68 (1.29, 2.19)**	**1.54 (1.16, 2.06)**	0.98 (0.78, 1.23)
Separated, divorced, or widowed	**5.10 (2.33, 11.13)**	1.90 (0.61, 5.93)	1.29 (0.51, 3.30)	**5.71 (2.56, 12.72)**	1.68 (0.53, 5.30)	1.26 (0.49, 3.26)
**Education**						
Less than HS	Ref	Ref	Ref	Ref	Ref	Ref
HS graduate/GED	1.14 (0.69, 1.90)	1.06 (0.63, 1.77)	1.05 (0.67, 1.65)	1.08 (0.65, 1.82)	1.21 (0.72, 2.05)	1.09 (0.69, 1.71)
Some college	1.23 (0.77, 1.98)	0.97 (0.60, 1.58)	1.22 (0.81, 1.84)	1.25 (0.77, 2.06)	1.26 (0.76, 2.08)	1.35 (0.88, 2.07)
Associate/technical	1.49 (0.91, 2.46)	1.09 (0.65, 1.82)	1.37 (0.88, 2.13)	**1.71 (1.00, 2.91)^**	1.62 (0.93, 2.82)	1.62 (1.01, 2.58)
Bachelor's degree	0.73 (0.46, 1.16)	0.59 (0.37, 0.95)	1.09 (0.73, 1.62)	0.87 (0.52, 1.47)	1.02 (0.59, 1.75)	1.36 (0.87, 2.12)
Beyond bachelor's	0.82 (0.51, 1.32)	0.57 (0.35, 0.92)	1.00 (0.67, 1.51)	1.00 (0.58, 1.71)	1.03 (0.59, 1.82)	1.26 (0.79, 2.00)
**Insurance**						
Commercial/military	Ref	Ref	Ref	Ref	Ref	Ref
Government	1.05 (0.84, 1.31)	**1.69 (1.35, 2.12)**	1.18 (0.99, 1.41)	**0.55 (0.41, 0.73)**	1.04 (0.77, 1.40)	1.07 (0.84, 1.36)
Self-pay/other	0.92 (0.55, 1.55)	0.84 (0.45, 1.57)	0.81 (0.52, 1.28)	0.64 (0.37, 1.10)	0.65 (0.34, 1.22)	0.77 (0.49, 1.22)

Definition of abbreviations: nuMoM2b= Nulliparous Pregnancy Outcomes Study: Monitoring Mothers-to-be; N=sample size; OR = odds ratio, CI = confidence interval; HS = high school; GED = general education development certificate.

^ When the confidence interval shown includes 1.0 due to rounding, this symbol indicates that the non-rounded CI excludes 1.0.
† Adjusted for baseline age and time from delivery to follow-up study visit.

© 2024 Kim M et al. *JAMA Network Open.*

eTable 2. Biological Measures of Hypertension and Metabolic Risk at First Pregnancy Visit, According to Short Sleep Duration Patterns

Characteristic at nuMoM2b enrollment	Overall, N=3,922	Never, N=1,977 (50.4%)	Persistent, N=565 (14.4%)	Resolved, N=448 (11.4%)	New, N=932 (23.8%)
**Body mass index**					
n	3870	1951	559	439	921
Mean (SD), kg/m^2	26.5 (6.4)	25.8 (5.8)	28.0 (7.5)	27.0 (6.6)	27.0 (6.6)
Category, n (%)					
<25 kg/m^2	2016 (52.1)	1114 (57.1)	246 (44.0)	212 (48.3)	444 (48.2)
25-<30 kg/m^2	954 (24.7)	464 (23.8)	142 (25.4)	109 (24.8)	239 (26.0)
≥30 kg/m^2	900 (23.3)	373 (19.1)	171 (30.6)	118 (26.9)	238 (25.8)
**Waist circumference**					
n	3843	1939	552	438	914
Mean (SD), cm	95.5 (14.7)	94.2 (13.4)	98.6 (16.2)	96.5 (15.2)	96.5 (15.2)
≥88 (non-Asian) or ≥80 cm (Asian), n (%)	2625 (68.3)	1278 (65.9)	409 (74.1)	298 (68.0)	640 (70.0)
**Blood pressure**					
N	3848	1936	557	439	916
SBP, mean (SD), mmHg	109.3 (10.8)	108.7 (10.6)	110.3 (11.1)	110.3 (11.4)	109.4 (10.4)
DBP, mean (SD), mmHg	67.3 (8.3)	67.0 (8.2)	68.0 (8.6)	67.2 (8.4)	67.4 (8.3)
SBP ≥130mmHg, DBP ≥80mmHg, or on antihypertensive medication n/N (%)	503/3846 (13.1)	224/1936 (11.6)	86/557 (15.4)	69/437 (15.8)	124/916 (13.5)
**Triglycerides**					
N	3821	1918	551	437	915
Mean (SD), mg/dl	127.0 (51.0)	127.0 (49.3)	129.3 (54.9)	127.6 (53.1)	125.1 (51.1)
≥150 mg/dl or on lipid-lowering medication, n/N (%)	1000/3817 (26.2)	498/1916 (26.0)	165/551 (29.9)	112/435 (25.7)	225/915 (24.6)
**HDL cholesterol**					
N	3820	1917	551	437	915
Mean (SD), mg/dl	72.7 (15.2)	72.9 (15.1)	72.0 (15.1)	72.6 (15.5)	72.8 (15.3)
<50 mg/dl or on HDL-raising medication, n/N (%)	168/3816 (4.4)	78/1915 (4.1)	29/551 (5.3)	18/435 (4.1)	43/915 (4.7)
**Fasting blood glucose**					
N	3821	1918	551	437	915

© 2024 Kim M et al. *JAMA Network Open.*

Characteristic at nuMoM2b enrollment	Overall, N=3,922	Never, N=1,977 (50.4%)	Persistent, N=565 (14.4%)	Resolved, N=448 (11.4%)	New, N=932 (23.8%)
Mean (SD), mg/dl	88.1 (15.7)	88.0 (15.5)	89.6 (15.4)	86.9 (16.7)	87.8 (15.6)
≥100 mg/dl or on glucose-lowering medication, n/N (%)	711/3815 (18.6)	337/1917 (17.6)	113/551 (20.5)	83/434 (19.1)	178/913 (19.5)
**Prevalent Metabolic syndrome, n/N (%)†**	371/3795 (9.8)	171/1907 (9.0)	64/548 (11.7)	43/430 (10.0)	93/910 (10.2)

Definition of abbreviations: nuMoM2b= Nulliparous Pregnancy Outcomes Study: Monitoring Mothers-to-be; N=sample size; n=number in category; SD=standard deviation; SBP = systolic blood pressure; DBP = diastolic blood pressure; HDL = high-density lipoprotein cholesterol; HHS=Heart Healthy Study.

† Metabolic syndrome is defined based on the presence of three of five of the following criteria: elevated waist circumference, elevated triglycerides or associated medication, elevated fasting glucose or associated medication, elevated blood pressure (SBP ≥130 or DBP ≥ 85) or associated medication, and reduced HDL cholesterol or associated medication.

© 2024 Kim M et al. *JAMA Network Open.*

eTable 3. Association of Short Sleep Duration Patterns with Incident Hypertension and Metabolic Syndrome 2-7 Years after Index Pregnancy

Short sleep duration pattern	Incident Hypertension			Incident Metabolic Syndrome		
	n / N (%)	Unadjusted OR (95% CI)	Adjusted OR (95% CI)†	n / N (%)	Unadjusted OR (95% CI)	Adjusted OR (95% CI)‡
**All participants**	599/3,336 (18.0)	-	-	447/3,373 (13.3)	-	-
**Never**	309/1,710 (18.1)	Ref	Ref	214/1,709 (12.5)	Ref	Ref
**Persistent**	92/469 (19.6)	1.11 (0.85, 1.43)	0.91 (0.69, 1.19)	86/475 (18.1)	**1.54 (1.17, 2.03)**	**1.60 (1.21, 2.11)**
**Resolved**	66/368 (17.9)	0.99 (0.74, 1.33)	0.92 (0.68, 1.25)	49/383 (12.8)	1.02 (0.74, 1.43)	1.00 (0.72, 1.40)
**New**	132/789 (16.7)	0.91 (0.73, 1.14)	0.83 (0.66, 1.05)	98/806 (12.2)	0.97 (0.75, 1.25)	0.99 (0.77, 1.28)

Definition of abbreviations: n = number in category; N = sample size; OR = odds ratio; CI = confidence interval; Ref = reference

† Adjusted for baseline age, early index pregnancy body mass index, and time from delivery to follow-up study visit.
‡ Adjusted for baseline age and time from delivery to follow-up study visit.

© 2024 Kim M et al. *JAMA Network Open.*

eTable 4. Association of Sleep-Disordered Breathing and Short Sleep Duration Patterns (defined as <7 hours) with Incident Hypertension and Metabolic Syndrome 2-7 Years after Index Pregnancy

Sleep-disordered breathing	Short sleep duration pattern	Incident Hypertension		Incident Metabolic Syndrome	
		Unadjusted OR (95% CI)	Adjusted OR (95% CI)[†]	Unadjusted OR (95% CI)	Adjusted OR (95% CI)[‡]
AHI ≥ 5 [§]	Never	Ref	Ref	Ref	Ref
	Persistent	2.71 (0.76, 9.58)	1.27 (0.33, 4.82)	4.83 (1.40, 16.68)	4.66 (1.33, 16.37)
	Resolved	--	--	2.23 (0.22, 22.54)	2.44 (0.24, 24.92)
	New	2.35 (0.57, 9.72)	1.23 (0.29, 5.22)	4.55 (1.26, 16.42)	4.20 (1.14, 15.48)
	Interaction	P-value = 0.3	P-value = 0.11	P-value = 0.9	P-value = 0.9
AHI < 5 [§]	Never	Ref	Ref	Ref	Ref
	Persistent	0.37 (0.10, 1.31)	0.79 (0.21, 2.99)	0.21 (0.06, 0.71)	0.21 (0.06, 0.75)
	Resolved	--	--	0.45 (0.04, 4.54)	0.41 (0.04, 4.19)
	New	0.43 (0.10, 1.76)	0.81 (0.19, 3.46)	0.22 (0.06, 0.79)	0.24 (0.06, 0.88)
	Interaction	P-value = 0.3	P-value = 0.1	P-value = 0.9	P-value = 0.9
ODI ≥ 5 [¶]	Never	Ref	Ref	Ref	Ref
	Persistent	2.71 (0.76, 9.58)	1.27 (0.33, 4.82)	4.83 (1.40, 16.68)	4.66 (1.33, 16.37)
	Resolved	--	--	2.23 (0.22, 22.54)	2.44 (0.24, 24.92)
	New	2.35 (0.57, 9.72)	1.23 (0.29, 5.22)	4.55 (1.26, 16.42)	4.20 (1.14, 15.48)
	Interaction	P-value = 0.3	P-value = 0.11	P-value = 0.9	P-value = 0.9
ODI < 5 [¶]	Never	Ref	Ref	Ref	Ref
	Persistent	0.37 (0.10, 1.31)	0.79 (0.21, 2.99)	0.21 (0.06, 0.71)	0.21 (0.06, 0.75)
	Resolved	--	--	0.45 (0.04, 4.54)	0.41 (0.04, 4.19)
	New	0.43 (0.10, 1.76)	0.81 (0.19, 3.46)	0.22 (0.06, 0.79)	0.24 (0.06, 0.88)
	Interaction	P-value = 0.3	P-value = 0.1	P-value = 0.9	P-value = 0.9

© 2024 Kim M et al. *JAMA Network Open.*

Definition of abbreviations: n = number in category; N = sample size; OR = odds ratio; CI = confidence interval; Ref = reference; AHI = Apnea-hypopnea index; ODI = Oxygen desaturation index.

† Adjusted for baseline age, early index pregnancy body mass index, and time from delivery to follow-up study visit.
‡ Adjusted for baseline age and time from delivery to follow-up study visit.
§ Apnea-hypopnea index (AHI) is the number of apneas and hypopneas per hour of estimated sleep, including all apneas plus hypopneas accompanied by ≥ 3% oxygen desaturation.
¶ Oxygen desaturation index (ODI) is the number of oxygen desaturations ≥ 3% from before the event baseline per hour of estimated sleep.

© 2024 Kim M et al. *JAMA Network Open.*

eTable 5. Association of nuMoM2b Sociodemographic Characteristics with Short Sleep Duration Patterns (short sleep defined as <6 hours)

Baseline Characteristics	Unadjusted Models			Adjusted Models†		
	Persistent vs Never, OR (95% CI)	Resolved vs Never, OR (95% CI)	New vs Never, OR (95% CI)	Persistent vs Never, OR (95% CI)	Resolved vs Never, OR (95% CI)	New vs Never, OR (95% CI)
**Race and ethnicity**						
White Non-Hispanic	Ref	Ref	Ref	Ref	Ref	Ref
Black Non-Hispanic	**3.10 (1.90, 5.05)**	**2.73 (1.90, 3.91)**	**2.86 (2.10, 3.89)**	1.73 (0.98, 3.04)	**1.68 (1.11, 2.55)**	**2.42 (1.68, 3.49)**
Hispanic	1.50 (0.86, 2.61)	**1.73 (1.20, 2.51)**	**1.61 (1.16, 2.23)**	1.03 (0.56, 1.88)	1.28 (0.85, 1.91)	**1.44 (1.01, 2.06)**
Asian	-	0.74 (0.27, 2.03)	1.22 (0.61, 2.45)	-	0.91 (0.33, 2.54)	1.34 (0.66, 2.72)
Other	1.43 (0.56, 3.63)	0.80 (0.35, 1.86)	1.38 (0.78, 2.44)	1.06 (0.41, 2.76)	0.64 (0.27, 1.50)	1.28 (0.72, 2.29)
**Marital status**						
Married	Ref	Ref	Ref	Ref	Ref	Ref
Never married	**3.53 (2.32, 5.36)**	**2.70 (2.03, 3.58)**	**1.72 (1.35, 2.19)**	**2.78 (1.61, 4.79)**	**2.18 (1.49, 3.19)**	1.07 (0.76, 1.50)
Separated, divorced, or widowed	**6.42 (1.87, 22.04)**	2.54 (0.76, 8.49)	0.96 (0.23, 4.05)	**5.47 (1.54, 19.41)**	2.34 (0.69, 7.98)	0.77 (0.18, 3.30)
**Education**						
Less than HS	Ref	Ref	Ref	Ref	Ref	Ref
HS graduate/GED	1.00 (0.43, 2.36)	1.37 (0.70, 2.71)	1.14 (0.64, 2.03)	1.02 (0.43, 2.43)	1.41 (0.71, 2.81)	1.18 (0.66, 2.13)
Some college	0.67 (0.29, 1.52)	0.95 (0.49, 1.83)	0.95 (0.55, 1.65)	0.79 (0.34, 1.86)	1.15 (0.59, 2.26)	1.07 (0.61, 1.88)
Associate/technical	1.17 (0.51, 2.67)	1.22 (0.62, 2.41)	0.74 (0.41, 1.36)	1.78 (0.74, 4.29)	1.77 (0.86, 3.63)	0.95 (0.50, 1.81)
Bachelor's degree	0.27 (0.11, 0.64)	0.60 (0.31, 1.16)	0.54 (0.31, 0.93)	0.48 (0.18, 1.28)	1.05 (0.51, 2.16)	0.72 (0.39, 1.35)
Beyond bachelor's	0.27 (0.11, 0.68)	0.38 (0.19, 0.77)	0.55 (0.32, 0.97)	0.55 (0.20, 1.55)	0.71 (0.32, 1.58)	0.77 (0.40, 1.46)
**Insurance**						
Commercial/military	Ref	Ref	Ref	Ref	Ref	Ref
Government	**1.85 (1.21, 2.83)**	**1.77 (1.31, 2.38)**	**1.66 (1.28, 2.14)**	0.66 (0.39, 1.11)	0.79 (0.53, 1.15)	0.96 (0.68, 1.35)
Self-pay/other	1.40 (0.50, 3.92)	1.14 (0.52, 2.49)	0.44 (0.16, 1.21)	0.74 (0.25, 2.15)	0.71 (0.31, 1.59)	**0.32 (0.11, 0.88)**

Definition of abbreviations: nuMoM2b= Nulliparous Pregnancy Outcomes Study: Monitoring Mothers-to-be; N=sample size; OR = odds ratio, CI = confidence interval; HS = high school; GED = general education development certificate.

^ When the confidence interval shown includes 1.0 due to rounding, this symbol indicates that the non-rounded CI excludes 1.0.
† Adjusted for baseline age and time from delivery to follow-up study visit.

© 2024 Kim M et al. *JAMA Network Open.*

eFigure1. Association of Short Sleep Duration Patterns (defined as <6 hours) with Incident Hypertension (HTN) and Metabolic Syndrome (MetS) 2-7 Years after Index Pregnancy

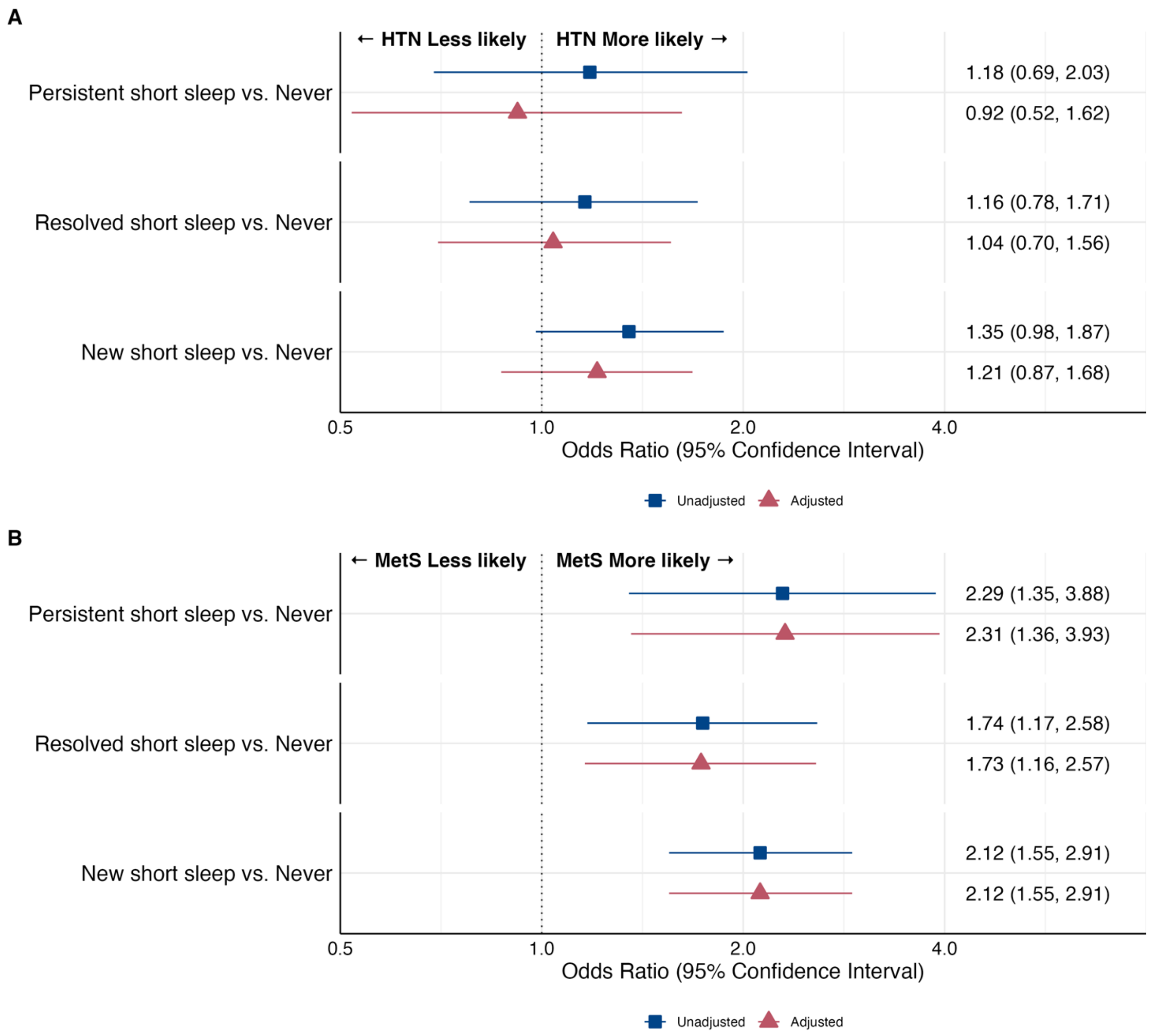

© 2024 Kim M et al. *JAMA Network Open.*

eFigure 1 Legend:

- **A.** With short sleep defined as less than 6 hours, associations between sleep duration patterns (referent: Never short sleep) with incident hypertension (HTN) are summarized as unadjusted (blue square) and adjusted (red triangle) odds ratios with 95% confidence intervals. Adjustment covariates included age and body mass index in early index pregnancy and time from delivery to follow-up study visit.
- **B.** With short sleep defined as less than 6 hours, associations between sleep duration patterns (referent: Never short sleep) with incident metabolic syndrome (MetS) are summarized as unadjusted (blue square) and adjusted (red triangle) odds ratios with 95% confidence intervals. Adjustment covariates included age in early index pregnancy and time from delivery to follow-up study visit.

© 2024 Kim M et al. *JAMA Network Open.*

eTable 6. Association of Short Sleep Duration Patterns (defined as <6 hours) with Incident Hypertension and Metabolic Syndrome 2-7 Years after Index Pregnancy

Short sleep duration pattern	Incident Hypertension			Incident Metabolic Syndrome		
	**n / N (%)**	**Unadjusted OR (95% CI)**	**Adjusted OR (95% CI)†**	**n / N (%)**	**Unadjusted OR (95% CI)**	**Adjusted OR (95% CI)‡**
**All participants**	599/3,336 (18.0)	-	-	447/3,373 (13.3)	-	-
**Never**	497/2,849 (17.4)	Ref	Ref	337/2,855 (11.8)	Ref	Ref
**Persistent**	17/85 (20.0)	1.18 (0.69, 2.03)	0.92 (0.52, 1.62)	19/81 (23.5)	**2.29 (1.35, 3.88)**	**2.31 (1.36, 3.93)**
**Resolved**	33/168 (19.6)	1.16 (0.78, 1.71)	1.04 (0.70, 1.56)	33/175 (18.9)	**1.74 (1.17, 2.58)**	**1.73 (1.16, 2.57)**
**New**	52/234 (22.2)	1.35 (0.98, 1.87)	1.21 (0.87, 1.68)	58/262 (22.1)	**2.12 (1.55, 2.91)**	**2.12 (1.55, 2.91)**

Definition of abbreviations: n = number in category; N = sample size; OR = odds ratio; CI = confidence interval; Ref = reference

† Adjusted for baseline age, early index pregnancy body mass index, and time from delivery to follow-up study visit.
‡ Adjusted for baseline age and time from delivery to follow-up study visit.

© 2024 Kim M et al. *JAMA Network Open.*

eTable 7. Association of Short Sleep Duration Patterns (defined as <7 hours) with Incident Hypertension and Metabolic Syndrome 2-7 Years after Index Pregnancy, Excluding Participants Who Reported Shift Work during Pregnancy

Short sleep duration pattern	Incident Hypertension			Incident Metabolic Syndrome		
	**n / N (%)**	**Unadjusted OR (95% CI)**	**Adjusted OR (95% CI)†**	**n / N (%)**	**Unadjusted OR (95% CI)**	**Adjusted OR (95% CI)‡**
**All participants**	430/2,479 (17.3)	-	-	320/2,488 (12.9)	-	-
**Never**	228/1,301 (17.5)	Ref	Ref	160/1,297 (12.3)	Ref	Ref
**Persistent**	57/314 (18.2)	1.04 (0.76, 1.44)	0.87 (0.62, 1.21)	51/309 (16.5)	**1.41 (1.00, 1.98)**	**1.48 (1.04, 2.09)**
**Resolved**	51/258 (19.8)	1.16 (0.83, 1.63)	1.08 (0.76, 1.53)	36/272 (13.2)	1.08 (0.74, 1.60)	1.07 (0.72, 1.58)
**New**	94/606 (15.5)	0.86 (0.66, 1.12)	0.80 (0.61, 1.05)	73/610 (12.0)	0.97 (0.72, 1.30)	1.01 (0.75, 1.35)

Analysis was conducted after excluding participants who reported afternoon, split, rotating, irregular/on-call, or night shift at Visit 1 or Visit 3 during the index pregnancy (n = 1,010).

Definition of abbreviations: n = number in category; N = sample size; OR = odds ratio; CI = confidence interval; Ref = reference

† Adjusted for baseline age, early index pregnancy body mass index, and time from delivery to follow-up study visit.
‡ Adjusted for baseline age and time from delivery to follow-up study visit.

© 2024 Kim M et al. *JAMA Network Open.*
