# Supplementary material for: Persistent Short Sleep Duration From Pregnancy to 2 to 7 Years After Delivery and Metabolic Health
Source: JAMA Netw Open. 2024 Dec 26;7(12):e2452204. doi: 10.1001/jamanetworkopen.2024.52204 (PMC11672157; doi:10.1001/jamanetworkopen.2024.52204)
Supplement: Supplement 2. — Nonauthor Collaborators. Eunice Kennedy Shriver National Institute of Child Health and Human Development NuMoM2b and National Heart, Lung, and Blood Institute NuMoM2b Heart Health Study Network [file jamanetwopen-e2452204-s002.pdf]

*Group Name(s): Eunice Kennedy Shriver National Institute of Child Health and Human Development NuMoM2b and National Heart, Lung, and Blood Institute NuMoM2b Heart Health Study Network							
***First Name and Middle Initial(s)**	***Last Name**	***Suffix (eg, Jr, III)**	Academic Degrees	Institution	Location (city, state/province, country)	Role or Contribution, eg, chair, principal investigator	Group (if more than 1 Group listed in the byline) and/or Subgroup (eg, Steering Committee)
Wendy	Dalton		RN	Case Western Reserve University - The MetroHealth System	Cleveland, OH	Study Coordinator	NICHD nuMoM2b network
Judette M.	Louis		MD	Case Western Reserve University - The MetroHealth System	Cleveland, OH	Co-investigator	NICHD nuMoM2b network
Brian M.	Mercer		MD	Case Western Reserve University - The MetroHealth System	Cleveland, OH	Site PI	NICHD nuMoM2b network
LuAnn	Polito		RN, JD	Case Western Reserve University - The MetroHealth System	Cleveland, OH	Study Coordinator	NICHD nuMoM2b network
Jay	Iams		MD	Case Western Reserve University - The MetroHealth System / The Ohio State University	Columbus, OH	Site PI	NICHD nuMoM2b network
Cheryl	Latimer		RN	Case Western Reserve University - The MetroHealth System / The Ohio State University	Columbus, OH	Study Coordinator	NICHD nuMoM2b network
Robert C.	Basner		MD	Columbia University	New York, NY	Co-investigator	NICHD nuMoM2b network
Michelle	DiVito		MSN, CCRC	Columbia University	New York, NY	Study Coordinator	NICHD nuMoM2b network
Karin	Fuchs		MD	Columbia University	New York, NY	Co-investigator	NICHD nuMoM2b network
Chia-Ling	Nhan-Chang		MD	Columbia University	New York, NY	Co-investigator	NICHD nuMoM2b network
Ameneh	Onativia		MD	Columbia University	New York, NY	Co-investigator	NICHD nuMoM2b network
Caroline	Torres		MD	Columbia University	New York, NY	Study Coordinator	NICHD nuMoM2b network
Ronald	Wapner		MD	Columbia University	New York, NY	Site PI	NICHD nuMoM2b network

***First Name and Middle Initial(s)**	***Last Name**	***Suffix (eg, Jr, III)**	Academic Degrees	Institution	Location (city, state/province, country)	Role or Contribution, eg, chair, principal investigator	Group (if more than 1 Group listed in the byline) and/or Subgroup (eg, Steering Committee)
Matthew K.	Hoffman		MD, MPH	Columbia University / Christiana Care	Wilmington, DE	Site PI	NICHD nuMoM2b network
Stephanie	Lynch		RN, BSN, CCRC	Columbia University / Christiana Care	Wilmington, DE	Study Coordinator	NICHD nuMoM2b network
Maurice	Davis		DHA, MPA, MHSA	*Eunice Kennedy Shriver* National Institute of Child Health and Human Development	Bethesda, MD, USA	Project Officer	NICHD nuMoM2b network
Uma M.	Reddy		MD, MPH	*Eunice Kennedy Shriver* National Institute of Child Health and Human Development	Bethesda, MD, USA	Project Scientist	NICHD nuMoM2b network
Marian	Willinger		PhD	*Eunice Kennedy Shriver* National Institute of Child Health and Human Development	Bethesda, MD, USA	Project Officer	NICHD nuMoM2b network
Daniel	Mobley		RPSGT	Harvard University, Brigham and Women's Hospital	Boston, MA	Co-investigator	NICHD nuMoM2b network
Susan	Redline		MD, MPH	Harvard University, Brigham and Women's Hospital	Boston, MA	Site PI	NICHD nuMoM2b network
Susan	Surovec		BA	Harvard University, Brigham and Women's Hospital	Boston, MA	Sleep Technician	NICHD nuMoM2b network
Julianne	Ulanski		BS	Harvard University, Brigham and Women's Hospital	Boston, MA	Sleep Technician	NICHD nuMoM2b network
Shannon	Barnes		RN, MSN	Indiana University	Indianapolis, IN	Study Coordinator	NICHD nuMoM2b network
Tatiana	Foroud		PhD	Indiana University	Indianapolis, IN	Co-investigator	NICHD nuMoM2b network
Catherine L.	McCormick		RN	Indiana University	Indianapolis, IN	Study Coordinator	NICHD nuMoM2b network
Emily	Perkins		BS, MA, CCRP	Indiana University	Indianapolis, IN	Study Coordinator	NICHD nuMoM2b network
Frank P.	Schubert		MD, MS	Indiana University	Indianapolis, IN	Co-investigator	NICHD nuMoM2b network

*First Name and Middle Initial(s)	*Last Name	*Suffix (eg, Jr, III)	Academic Degrees	Institution	Location (city, state/province, country)	Role or Contribution, eg, chair, principal investigator	Group (if more than 1 Group listed in the byline) and/or Subgroup (eg, Steering Committee)
Alicia	Winters		BS	Indiana University	Indianapolis, IN	Study Coordinator	NICHD nuMoM2b network
Aaron	Laposky		PhD	National Heart, Lung, and Blood Institute	Bethesda, MD, USA	Project Officer	NICHD nuMoM2b network
Peggy	Campbell		RN, BSN, CCRC	Northwestern University	Chicago, IL	Study Coordinator	NICHD nuMoM2b network
Alan M.	Peaceman		MD	Northwestern University	Chicago, IL	Co-investigator	NICHD nuMoM2b network
Jessica S.	Shepard		MPH	Northwestern University	Chicago, IL	Study Coordinator	NICHD nuMoM2b network
Crystal N.	Williams		BA	Northwestern University	Chicago, IL	Study Coordinator	NICHD nuMoM2b network
Barbara V.	Alexander		MSPH	RTI International	Research Triangle Park, NC	Statistician	NICHD nuMoM2b network
Martha J.	DeCain		BS	RTI International	Research Triangle Park, NC	Project Manager	NICHD nuMoM2b network
Holly L.	Franklin		MPH	RTI International	Research Triangle Park, NC	Study Coordinator	NICHD nuMoM2b network
Christopher	Griggs		BS	RTI International	Research Triangle Park, NC	Research Informatician	NICHD nuMoM2b network
Tommy E.	Holder	Jr.	BS	RTI International	Research Triangle Park, NC	Statistician	NICHD nuMoM2b network
Shannon	Hunter		MS	RTI International	Research Triangle Park, NC	Statistician	NICHD nuMoM2b network
Matthew A.	Koch		MD, PhD	RTI International	Research Triangle Park, NC	Co-investigator	NICHD nuMoM2b network
Deborah W.	McFadden		MBA	RTI International	Research Triangle Park, NC	Project Manager	NICHD nuMoM2b network
Corette B.	Parker		DrPH	RTI International	Research Triangle Park, NC	Site PI	NICHD nuMoM2b network

*First Name and Middle Initial(s)	*Last Name	*Suffix (eg, Jr, III)	Academic Degrees	Institution	Location (city, state/province, country)	Role or Contribution, eg, chair, principal investigator	Group (if more than 1 Group listed in the byline) and/or Subgroup (eg, Steering Committee)
Venkat	Yetukuri		MS	RTI International	Research Triangle Park, NC	Research Informatician	NICHD nuMoM2b network
Judith H.	Chung		MD, PhD	University of California - Irvine	Irvine, CA	Co-investigator	NICHD nuMoM2b network
Michael P.	Nageotte		MD	University of California - Irvine	Irvine, CA	Co-investigator	NICHD nuMoM2b network
Valerie	Pham		RDMS	University of California - Irvine	Irvine, CA	Sonographer	NICHD nuMoM2b network
Manuel	Porto		MD	University of California - Irvine	Irvine, CA	Study Coordinator	NICHD nuMoM2b network
Pamela J.	Rumney		RNC, CCRC	University of California - Irvine	Irvine, CA	Study Coordinator	NICHD nuMoM2b network
Pathik D.	Wadhwa		MD, PhD	University of California - Irvine	Irvine, CA	Co-investigator	NICHD nuMoM2b network
Deborah A.	Wing		MD	University of California - Irvine	Irvine, CA	Site PI	NICHD nuMoM2b network
Brittany	Araujo		BS	University of Pennsylvania	Philadelphia, PA	Study Coordinator	NICHD nuMoM2b network
Michal	Elovitz		MD	University of Pennsylvania	Philadelphia, PA	Co-investigator	NICHD nuMoM2b network
Jack	Ludmir		MD	University of Pennsylvania	Philadelphia, PA	Co-investigator	NICHD nuMoM2b network
Samuel	Parry		MD	University of Pennsylvania	Philadelphia, PA	Site PI	NICHD nuMoM2b network
Mary	Peters		BA, MPH	University of Pennsylvania	Philadelphia, PA	Study Coordinator	NICHD nuMoM2b network
Grace	Pien		MD, MSCE	University of Pennsylvania	Philadelphia, PA	Co-investigator	NICHD nuMoM2b network
Melissa	Bickus		RN, BS	University of Pittsburgh / Magee-Women's Hospital	Pittsburgh, PA	Study Coordinator	NICHD nuMoM2b network

*First Name and Middle Initial(s)	*Last Name	*Suffix (eg, Jr, III)	Academic Degrees	Institution	Location (city, state/province, country)	Role or Contribution, eg, chair, principal investigator	Group (if more than 1 Group listed in the byline) and/or Subgroup (eg, Steering Committee)
Steve N.	Caritis		MD	University of Pittsburgh / Magee-Women's Hospital	Pittsburgh, PA	Co-investigator	NICHD nuMoM2b network
Ashi R.	Daftary		MD	University of Pittsburgh / Magee-Women's Hospital	Pittsburgh, PA	Co-investigator	NICHD nuMoM2b network
Stephen P.	Emery		MD	University of Pittsburgh / Magee-Women's Hospital	Pittsburgh, PA	Co-investigator	NICHD nuMoM2b network
Francesca L.	Facco		MD	University of Pittsburgh / Magee-Women's Hospital	Pittsburgh, PA	Co-investigator	NICHD nuMoM2b network
Hyagriv N.	Simhan		MD, MSCR	University of Pittsburgh / Magee-Women's Hospital	Pittsburgh, PA	Site PI	NICHD nuMoM2b network
Paul D.	Speer		MD	University of Pittsburgh / Magee-Women's Hospital	Pittsburgh, PA	Co-investigator	NICHD nuMoM2b network
George R.	Saade		MD	University of Texas Medical Branch	Galveston, TX	Steering Committee chair	NICHD nuMoM2b network
Kelly	Christensen		RN	University of Utah	Salt Lake City, UT	Study Coordinator	NICHD nuMoM2b network
M. Sean	Esplin		MD	University of Utah	Salt Lake City, UT	Co-investigator	NICHD nuMoM2b network
Linda	Meadows		RN	University of Utah	Salt Lake City, UT	Study Coordinator	NICHD nuMoM2b network
Valerie	Morby		RN	University of Utah	Salt Lake City, UT	Study Coordinator	NICHD nuMoM2b network
Julie	Postma		RN	University of Utah	Salt Lake City, UT	Study Coordinator	NICHD nuMoM2b network
Melanie	Williams		RN	University of Utah	Salt Lake City, UT	Study Coordinator	NICHD nuMoM2b network
Ashish	Aneja		MD	Case Western Reserve University - The MetroHealth System	Cleveland, OH	Co-investigator	NHLBI nuMoM2b Heart Health Study network
Kelly	Gibson		MD	Case Western Reserve University - The MetroHealth System	Cleveland, OH	Co-investigator	NHLBI nuMoM2b Heart Health Study network

*First Name and Middle Initial(s)	*Last Name	*Suffix (eg, Jr, III)	Academic Degrees	Institution	Location (city, state/province, country)	Role or Contribution, eg, chair, principal investigator	Group (if more than 1 Group listed in the byline) and/or Subgroup (eg, Steering Committee)
Judette M.	Louis		MD	Case Western Reserve University - The MetroHealth System	Cleveland, OH	Co-investigator	NHLBI nuMoM2b Heart Health Study network
Brian M.	Mercer		MD	Case Western Reserve University - The MetroHealth System	Cleveland, OH	Site PI	NHLBI nuMoM2b Heart Health Study network
Jessica	Pippen		MD	Case Western Reserve University - The MetroHealth System	Cleveland, OH	Site PI	NHLBI nuMoM2b Heart Health Study network
LuAnn	Polito		RN, JD	Case Western Reserve University - The MetroHealth System	Cleveland, OH	Study Coordinator	NHLBI nuMoM2b Heart Health Study network
Bonnie	Rosolowski		BS, CCRC	Case Western Reserve University - The MetroHealth System	Cleveland, OH	Study Coordinator	NHLBI nuMoM2b Heart Health Study network
Laniece	Thomas		MPH	Case Western Reserve University - The MetroHealth System	Cleveland, OH	Study Coordinator	NHLBI nuMoM2b Heart Health Study network
C. Noel	Bairey Merz		MD	Cedars-Sinai Medical Center	Los Angeles, CA	Site PI	NHLBI nuMoM2b Heart Health Study network
Natalie	Bello		MD, MPH	Cedars-Sinai Medical Center	Los Angeles, CA	Ancillary Study Investigator	NHLBI nuMoM2b Heart Health Study network
Ian	Brower		BS	Cedars-Sinai Medical Center	Los Angeles, CA	Biorepository Technician	NHLBI nuMoM2b Heart Health Study network
Maria	Marroquin		BS	Cedars-Sinai Medical Center	Los Angeles, CA	Biorepository Technician	NHLBI nuMoM2b Heart Health Study network
Jenna	Maughan		BA	Cedars-Sinai Medical Center	Los Angeles, CA	Biospecimen Manager	NHLBI nuMoM2b Heart Health Study network
Nathan	Sela		BS	Cedars-Sinai Medical Center	Los Angeles, CA	Biorepository Technician	NHLBI nuMoM2b Heart Health Study network
Brandon	Wanke		BS	Cedars-Sinai Medical Center	Los Angeles, CA	Biorepository Technician	NHLBI nuMoM2b Heart Health Study network
Eileen	Yu		BS	Cedars-Sinai Medical Center	Los Angeles, CA	Biorepository Technician	NHLBI nuMoM2b Heart Health Study network
Casandra	Almonte		MD	Columbia University	New York, NY	Lead Coordinator	NHLBI nuMoM2b Heart Health Study network

*First Name and Middle Initial(s)	*Last Name	*Suffix (eg, Jr, III)	Academic Degrees	Institution	Location (city, state/province, country)	Role or Contribution, eg, chair, principal investigator	Group (if more than 1 Group listed in the byline) and/or Subgroup (eg, Steering Committee)
Cande	Ananth		PhD, MPH	Columbia University	New York, NY	Co-investigator	NHLBI nuMoM2b Heart Health Study network
Veronica	Barcelona		PhD, MSN, PHNA-BC, RN	Columbia University	New York, NY	Co-investigator	NHLBI nuMoM2b Heart Health Study network
Robert C.	Basner		MD	Columbia University	New York, NY	Co-investigator	NHLBI nuMoM2b Heart Health Study network
Natalie	Bello		MD	Columbia University	New York, NY	Ancillary Study Investigator	NHLBI nuMoM2b Heart Health Study network
Whitney	Booker		MD	Columbia University	New York, NY	Investigator	NHLBI nuMoM2b Heart Health Study network
Jeremy	Chiu		MS	Columbia University	New York, NY	Study Coordinator	NHLBI nuMoM2b Heart Health Study network
Kirsten	Cleary		MD	Columbia University	New York, NY	Co-investigator	NHLBI nuMoM2b Heart Health Study network
Nathalie	De La Cruz		MD	Columbia University	New York, NY	Study Coordinator	NHLBI nuMoM2b Heart Health Study network
Michelle	DiVito		MSN, CCRC	Columbia University	New York, NY	Study Coordinator	NHLBI nuMoM2b Heart Health Study network
Brandy	Firman		BSPH	Columbia University	New York, NY	Study Coordinator	NHLBI nuMoM2b Heart Health Study network
Karin	Fuchs		MD	Columbia University	New York, NY	Co-investigator	NHLBI nuMoM2b Heart Health Study network
Yessenia	Gutierrez		Medical Assistant	Columbia University	New York, NY	Study Coordinator	NHLBI nuMoM2b Heart Health Study network
Noora	Haghighi		MS	Columbia University	New York, NY	Coordinator	NHLBI nuMoM2b Heart Health Study network
Carla	Haro		BS	Columbia University	New York, NY	Study Coordinator	NHLBI nuMoM2b Heart Health Study network

***First Name and Middle Initial(s)**	***Last Name**	***Suffix (eg, Jr, III)**	Academic Degrees	Institution	Location (city, state/province, country)	Role or Contribution, eg, chair, principal investigator	Group (if more than 1 Group listed in the byline) and/or Subgroup (eg, Steering Committee)
Jennifer	Haythe		MD	Columbia University	New York, NY	Co-investigator	NHLBI nuMoM2b Heart Health Study network
Tal	Korem		PhD	Columbia University	New York, NY	Ancillary Study Investigator	NHLBI nuMoM2b Heart Health Study network
Eliza	Miller		MD, MS	Columbia University	New York, NY	Ancillary Study Investigator	NHLBI nuMoM2b Heart Health Study network
Chia-Ling	Nhan-Chang		MD	Columbia University	New York, NY	Co-investigator	NHLBI nuMoM2b Heart Health Study network
Ameneh	Onativia		MD	Columbia University	New York, NY	Co-investigator	NHLBI nuMoM2b Heart Health Study network
Belgica	Peguero		AS, Medical Assistant	Columbia University	New York, NY	Study Coordinator	NHLBI nuMoM2b Heart Health Study network
Uma	Reddy		MD	Columbia University	New York, NY	Site PI	NHLBI nuMoM2b Heart Health Study network
Jeimy	Rosado Feliz		MS	Columbia University	New York, NY	Lead Coordinator	NHLBI nuMoM2b Heart Health Study network
Caroline	Torres		MD	Columbia University	New York, NY	Study Coordinator	NHLBI nuMoM2b Heart Health Study network
Ronald	Wapner		MD	Columbia University	New York, NY	Site PI	NHLBI nuMoM2b Heart Health Study network
Qi	Yan		MS, PhD	Columbia University	New York, NY	Co-investigator	NHLBI nuMoM2b Heart Health Study network
Matthew K.	Hoffman		MD, MPH	Columbia University / Christiana Care	Wilmington, DE	Site PI	NHLBI nuMoM2b Heart Health Study network
Carrie	Kitto			Columbia University / Christiana Care	New York, NY	Lead Coordinator	NHLBI nuMoM2b Heart Health Study network
Stephanie	Lynch		RN, BSN, CCRC	Columbia University / Christiana Care	Wilmington, DE	Study Coordinator	NHLBI nuMoM2b Heart Health Study network
Jennifer	Nava			Columbia University / Christiana Care	New York, NY	Study Coordinator	NHLBI nuMoM2b Heart Health Study network

***First Name and Middle Initial(s)**	***Last Name**	***Suffix (eg, Jr, III)**	Academic Degrees	Institution	Location (city, state/province, country)	Role or Contribution, eg, chair, principal investigator	Group (if more than 1 Group listed in the byline) and/or Subgroup (eg, Steering Committee)
Amy	Staples		RN, MSN	Columbia University / Christiana Care	New York, NY	Study Coordinator	NHLBI nuMoM2b Heart Health Study network
Tetsuya	Kawakita		MD	Eastern Virginia Medical School	Norfolk, VA	Co-investigator	NHLBI nuMoM2b Heart Health Study network
George R.	Saade		MD	Eastern Virginia Medical School	Galveston, TX	PI	NHLBI nuMoM2b Heart Health Study network
Maurice	Davis		DHA, MPA, MHSA	*Eunice Kennedy Shriver* National Institute of Child Health and Human Development	Bethesda, MD, USA	Project Officer	NHLBI nuMoM2b Heart Health Study network
Cathy	Spong		MD	*Eunice Kennedy Shriver* National Institute of Child Health and Human Development	Bethesda, MD, USA	Project Officer	NHLBI nuMoM2b Heart Health Study network
Michael	Honigberg		PhD	Harvard University	Cambridge, MA	Co-investigator	NHLBI nuMoM2b Heart Health Study network
Daniel	Mobley		RPSGT	Harvard University, Brigham and Women's Hospital	Boston, MA	Co-investigator	NHLBI nuMoM2b Heart Health Study network
Susan	Redline		MD, MPH	Harvard University, Brigham and Women's Hospital	Boston, MA	Site PI	NHLBI nuMoM2b Heart Health Study network
Julianne	Ulanski		BS	Harvard University, Brigham and Women's Hospital	Boston, MA	Sleep Technician	NHLBI nuMoM2b Heart Health Study network
Erica-Jane	Maro		BA	Indiana University	Indianapolis, IN	Study Coordinator	NHLBI nuMoM2b Heart Health Study network
Jessica	Roby-Fout		BS, CCRP	Indiana University	Indianapolis, IN	Study Coordinator	NHLBI nuMoM2b Heart Health Study network
Frank P.	Schubert		MD, MS	Indiana University	Indianapolis, IN	Co-investigator	NHLBI nuMoM2b Heart Health Study network
Christina	Scifres		MD	Indiana University	Indianapolis, IN	Co-investigator	NHLBI nuMoM2b Heart Health Study network
Hannah	Shockley		RN	Indiana University	Indianapolis, IN	Study Coordinator	NHLBI nuMoM2b Heart Health Study network

***First Name and Middle Initial(s)**	***Last Name**	***Suffix (eg, Jr, III)**	Academic Degrees	Institution	Location (city, state/province, country)	Role or Contribution, eg, chair, principal investigator	Group (if more than 1 Group listed in the byline) and/or Subgroup (eg, Steering Committee)
Ligia	Vasquez-Hout		MS	Indiana University	Indianapolis, IN	Lead Coordinator	NHLBI nuMoM2b Heart Health Study network
Haley	Whipps		BS	Indiana University	Indianapolis, IN	Study Coordinator	NHLBI nuMoM2b Heart Health Study network
Brittany	Yeley		RN	Indiana University	Indianapolis, IN	Study Coordinator	NHLBI nuMoM2b Heart Health Study network
Anum	Minhas		MD; MS	Johns Hopkins University	Baltimore, MD	Co-investigator	NHLBI nuMoM2b Heart Health Study network
Aaron	Laposky		PhD	National Heart, Lung, and Blood Institute	Bethesda, MD, USA	Project Officer	NHLBI nuMoM2b Heart Health Study network
Victoria L.	Pemberton		RNC, MS, CCRC	National Heart, Lung, and Blood Institute	Bethesda, MD, USA	Project Scientist	NHLBI nuMoM2b Heart Health Study network
Michael	Twery		PhD	National Heart, Lung, and Blood Institute	Bethesda, MD, USA	Program Scientist	NHLBI nuMoM2b Heart Health Study network
Jasmina	Varagic		MD, PhD	National Heart, Lung, and Blood Institute	Bethesda, MD, USA	Project Scientist	NHLBI nuMoM2b Heart Health Study network
Peggy	Campbell		RN, BSN, CCRC	Northwestern University	Chicago, IL	Study Coordinator	NHLBI nuMoM2b Heart Health Study network
Kiarri	Kershaw		PhD, MPH	Northwestern University	Chicago, IL	Ancillary Study Investigator	NHLBI nuMoM2b Heart Health Study network
Sadiya	Khan		MD, MSc	Northwestern University	Chicago, IL	Ancillary Study Investigator	NHLBI nuMoM2b Heart Health Study network
Elizabeth	Rangel		RN, BSN, CCRC	Northwestern University	Chicago, IL	Study Coordinator	NHLBI nuMoM2b Heart Health Study network
McKenzie	Jancsura		PhD, RN	Ohio State University Medical Center	Columbus, OH	Co-investigator	NHLBI nuMoM2b Heart Health Study network
Courtney	Lynch		PhD, MPH	Ohio State University Medical Center	Columbus, OH	Co-investigator	NHLBI nuMoM2b Heart Health Study network
Kartik	Venkatesh		MD PhD	Ohio State University Medical Center	Columbus, OH	Co-investigator	NHLBI nuMoM2b Heart Health Study network

***First Name and Middle Initial(s)**	***Last Name**	***Suffix (eg, Jr, III)**	Academic Degrees	Institution	Location (city, state/province, country)	Role or Contribution, eg, chair, principal investigator	Group (if more than 1 Group listed in the byline) and/or Subgroup (eg, Steering Committee)
Jiqiang	Wu		MSc	Ohio State University Medical Center	Columbus, OH	Statistician	NHLBI nuMoM2b Heart Health Study network
Carla	Bann		PhD	RTI International	Research Triangle Park, NC	Statistician	NHLBI nuMoM2b Heart Health Study network
Shannon	Barnes		RN, MSN, BA, CCRP	RTI International	Research Triangle Park, NC	Project Manager	NHLBI nuMoM2b Heart Health Study network
Benjamin	Carper		MS	RTI International	Research Triangle Park, NC	Statistician	NHLBI nuMoM2b Heart Health Study network
Emma	Crenshaw		BSPH	RTI International	Research Triangle Park, NC	Statistician	NHLBI nuMoM2b Heart Health Study network
Martha J.	DeCain		BS	RTI International	Research Triangle Park, NC	Project Manager	NHLBI nuMoM2b Heart Health Study network
Brian	Erman		MS	RTI International	Research Triangle Park, NC	Technologist	NHLBI nuMoM2b Heart Health Study network
Katie	Fain		MBA	RTI International	Research Triangle Park, NC	Project Manager	NHLBI nuMoM2b Heart Health Study network
Carl	Fisher		BS	RTI International	Research Triangle Park, NC	Research Informatician	NHLBI nuMoM2b Heart Health Study network
Holly L.	Franklin		MPH	RTI International	Research Triangle Park, NC	Study Coordinator	NHLBI nuMoM2b Heart Health Study network
Jace	Gilbert		MS	RTI International	Research Triangle Park, NC	Statistician	NHLBI nuMoM2b Heart Health Study network
Selen	Gizlice		BS	RTI International	Research Triangle Park, NC	Statistician	NHLBI nuMoM2b Heart Health Study network
Christopher	Griggs		BS	RTI International	Research Triangle Park, NC	Research Informatician	NHLBI nuMoM2b Heart Health Study network
Sean	Hanlon		PhD	RTI International	Research Triangle Park, NC	Technologist	NHLBI nuMoM2b Heart Health Study network
Mallory	Harris		MS	RTI International	Research Triangle Park, NC	Project Manager	NHLBI nuMoM2b Heart Health Study network

***First Name and Middle Initial(s)**	***Last Name**	***Suffix (eg, Jr, III)**	Academic Degrees	Institution	Location (city, state/province, country)	Role or Contribution, eg, chair, principal investigator	Group (if more than 1 Group listed in the byline) and/or Subgroup (eg, Steering Committee)
Joey	Hoellerich		BS	RTI International	Research Triangle Park, NC	Technologist	NHLBI nuMoM2b Heart Health Study network
Tommy E.	Holder	Jr.	BS	RTI International	Research Triangle Park, NC	Statistician	NHLBI nuMoM2b Heart Health Study network
Shannon	Hunter		MS	RTI International	Research Triangle Park, NC	Statistician	NHLBI nuMoM2b Heart Health Study network
Karen	Kesler		PhD	RTI International	Research Triangle Park, NC	Co-investigator	NHLBI nuMoM2b Heart Health Study network
Nick	Kinsey		BA	RTI International	Research Triangle Park, NC	Research Informatician	NHLBI nuMoM2b Heart Health Study network
Matthew A.	Koch		MD, PhD	RTI International	Research Triangle Park, NC	Co-investigator	NHLBI nuMoM2b Heart Health Study network
David	Leblond		BS	RTI International	Research Triangle Park, NC	Technologist	NHLBI nuMoM2b Heart Health Study network
Derek	Marsh		MS	RTI International	Research Triangle Park, NC	Statistician	NHLBI nuMoM2b Heart Health Study network
Deborah W.	McFadden		MBA	RTI International	Research Triangle Park, NC	Project Manager	NHLBI nuMoM2b Heart Health Study network
Taya	McMillan		BS	RTI International	Research Triangle Park, NC	Project Manager	NHLBI nuMoM2b Heart Health Study network
Heather	Meier		MS	RTI International	Research Triangle Park, NC	Statistician	NHLBI nuMoM2b Heart Health Study network
Janet	Moore		MS	RTI International	Research Triangle Park, NC	Statistician	NHLBI nuMoM2b Heart Health Study network
Eleanor	Nielson		MS	RTI International	Research Triangle Park, NC	Statistician	NHLBI nuMoM2b Heart Health Study network
Corette B.	Parker		DrPH	RTI International	Research Triangle Park, NC	PI	NHLBI nuMoM2b Heart Health Study network
Callie	Riggs		MBA	RTI International	Research Triangle Park, NC	Project Manager	NHLBI nuMoM2b Heart Health Study network

*First Name and Middle Initial(s)	*Last Name	*Suffix (eg, Jr, III)	Academic Degrees	Institution	Location (city, state/province, country)	Role or Contribution, eg, chair, principal investigator	Group (if more than 1 Group listed in the byline) and/or Subgroup (eg, Steering Committee)
Evan	Rhodes		BS	RTI International	Research Triangle Park, NC	Statistician	NHLBI nuMoM2b Heart Health Study network
Sridevi	Sattaluri		MS	RTI International	Secunderabad, India	Research Informatician	NHLBI nuMoM2b Heart Health Study network
Emily W.	Thomas		MS	RTI International	Research Triangle Park, NC	Research Informatician	NHLBI nuMoM2b Heart Health Study network
Vanessa	Thorsten		MS	RTI International	Arvada, CO	Statistician	NHLBI nuMoM2b Heart Health Study network
Nathan	Vandergrift		PhD	RTI International	Research Triangle Park, NC	Statistician	NHLBI nuMoM2b Heart Health Study network
Roger	Williams		MA, MPH	RTI International	Research Triangle Park, NC	Project Manager	NHLBI nuMoM2b Heart Health Study network
Venkat	Yetukuri		MS	RTI International	Research Triangle Park, NC	Research Informatician	NHLBI nuMoM2b Heart Health Study network
Yii-Der (Ida)	Chen		PhD	The Lundquist Institute	Los Angeles, CA	Site PI	NHLBI nuMoM2b Heart Health Study network
Judith H.	Chung		MD, PhD	University of California - Irvine	Irvine, CA	Site PI	NHLBI nuMoM2b Heart Health Study network
Phuong Linh L.	Huỳnh		MPH	University of California - Irvine	Irvine, CA	Lead Coordinator	NHLBI nuMoM2b Heart Health Study network
Jin Kyung	Kim		MD, MPH	University of California - Irvine	Irvine, CA	Co-investigator	NHLBI nuMoM2b Heart Health Study network
Manuel	Porto		MD	University of California - Irvine	Irvine, CA	Co-investigator	NHLBI nuMoM2b Heart Health Study network
Pamela J.	Rumney		RNC, CCRC	University of California - Irvine	Irvine, CA	Study Coordinator	NHLBI nuMoM2b Heart Health Study network
Nikka	Shahrokni		BS	University of California - Irvine	Irvine, CA	Student	NHLBI nuMoM2b Heart Health Study network
Lizette	Spiers		CRC	University of California - Irvine	Irvine, CA	Study Coordinator	NHLBI nuMoM2b Heart Health Study network

***First Name and Middle Initial(s)**	***Last Name**	***Suffix (eg, Jr, III)**	Academic Degrees	Institution	Location (city, state/province, country)	Role or Contribution, eg, chair, principal investigator	Group (if more than 1 Group listed in the byline) and/or Subgroup (eg, Steering Committee)
Amanda	Thornton		BS	University of California - Irvine	Irvine, CA	Study Coordinator	NHLBI nuMoM2b Heart Health Study network
Deborah A.	Wing		MD	University of California - Irvine	Irvine, CA	Site PI	NHLBI nuMoM2b Heart Health Study network
Jesi	Zavala		BS	University of California - Irvine	Irvine, CA	Study Coordinator	NHLBI nuMoM2b Heart Health Study network
Abbi	Lane		PhD	University of Michigan	Ann Arbor, Michigan	Ancillary Study Investigator	NHLBI nuMoM2b Heart Health Study network
Caroline	Huang		BS, MPH	University of Pennsylvania	Philadelphia, PA	Lead Coordinator	NHLBI nuMoM2b Heart Health Study network
Andrea	Kozai		PhD	University of Pennsylvania	Philadelphia, PA	Co-investigator	NHLBI nuMoM2b Heart Health Study network
Lisa	Levine		MD	University of Pennsylvania	Philadelphia, PA	Site PI	NHLBI nuMoM2b Heart Health Study network
Jennifer	Lewey		MD, MPH	University of Pennsylvania	Philadelphia, PA	Co-investigator	NHLBI nuMoM2b Heart Health Study network
Meaghan	McCabe		MPH	University of Pennsylvania	Philadelphia, PA	Lead Coordinator	NHLBI nuMoM2b Heart Health Study network
Maria	Monterroso		BA	University of Pennsylvania	Philadelphia, PA	Study Coordinator	NHLBI nuMoM2b Heart Health Study network
Nadia	Ngom		BA	University of Pennsylvania	Philadelphia, PA	Study Coordinator	NHLBI nuMoM2b Heart Health Study network
Samuel	Parry		MD	University of Pennsylvania	Philadelphia, PA	Co-investigator	NHLBI nuMoM2b Heart Health Study network
Grace	Pien		MD, MSCE	University of Pennsylvania	Philadelphia, PA	Site PI	NHLBI nuMoM2b Heart Health Study network
Keri	Simonette		BS	University of Pennsylvania	Philadelphia, PA	Study Coordinator	NHLBI nuMoM2b Heart Health Study network
Saisahana	Subburaj		BA, ABSN, MSN	University of Pennsylvania	Philadelphia, PA	Study Coordinator	NHLBI nuMoM2b Heart Health Study network

***First Name and Middle Initial(s)**	***Last Name**	***Suffix (eg, Jr, III)**	Academic Degrees	Institution	Location (city, state/province, country)	Role or Contribution, eg, chair, principal investigator	Group (if more than 1 Group listed in the byline) and/or Subgroup (eg, Steering Committee)
Fatoumata	Sy		BS	University of Pennsylvania	Philadelphia, PA	Study Coordinator	NHLBI nuMoM2b Heart Health Study network
Melissa	Bickus		RN, BS	University of Pittsburgh / Magee-Women's Hospital	Pittsburgh, PA	Study Coordinator	NHLBI nuMoM2b Heart Health Study network
Lisa	Bodnar		PhD	University of Pittsburgh / Magee-Women's Hospital	Pittsburgh, PA	Co-investigator	NHLBI nuMoM2b Heart Health Study network
Samantha	Bryan		MPH	University of Pittsburgh / Magee-Women's Hospital	Pittsburgh, PA	Lead Coordinator	NHLBI nuMoM2b Heart Health Study network
Steve N.	Caritis		MD	University of Pittsburgh / Magee-Women's Hospital	Pittsburgh, PA	Co-investigator	NHLBI nuMoM2b Heart Health Study network
Janet	Catov		PhD	University of Pittsburgh / Magee-Women's Hospital	Pittsburgh, PA	Site PI	NHLBI nuMoM2b Heart Health Study network
Christiana	Ekekwe		RN	University of Pittsburgh / Magee-Women's Hospital	Pittsburgh, PA	Study Coordinator	NHLBI nuMoM2b Heart Health Study network
Abigail	Evans		BS	University of Pittsburgh / Magee-Women's Hospital	Pittsburgh, PA	Study Coordinator	NHLBI nuMoM2b Heart Health Study network
Alisse	Hauspurg		MD	University of Pittsburgh / Magee-Women's Hospital	Pittsburgh, PA	Co-investigator	NHLBI nuMoM2b Heart Health Study network
Lacey	Heinsberg		PhD, RN	University of Pittsburgh / Magee-Women's Hospital	Pittsburgh, PA	Co-investigator	NHLBI nuMoM2b Heart Health Study network
Kimberly	Huber		MPH, CCRC	University of Pittsburgh / Magee-Women's Hospital	Pittsburgh, PA	Study Coordinator	NHLBI nuMoM2b Heart Health Study network
Michele	Levine		PhD	University of Pittsburgh / Magee-Women's Hospital	Pittsburgh, PA	Co-investigator	NHLBI nuMoM2b Heart Health Study network
Victoria	Lopata		BSN	University of Pittsburgh / Magee-Women's Hospital	Pittsburgh, PA	Study Coordinator	NHLBI nuMoM2b Heart Health Study network
Christofer	Price		BA	University of Pittsburgh / Magee-Women's Hospital	Pittsburgh, PA	Study Coordinator	NHLBI nuMoM2b Heart Health Study network
Mitali	Ray		PhD	University of Pittsburgh / Magee-Women's Hospital	Pittsburgh, PA	Ancillary Study Investigator	NHLBI nuMoM2b Heart Health Study network

***First Name and Middle Initial(s)**	***Last Name**	***Suffix (eg, Jr, III)**	Academic Degrees	Institution	Location (city, state/province, country)	Role or Contribution, eg, chair, principal investigator	Group (if more than 1 Group listed in the byline) and/or Subgroup (eg, Steering Committee)
Hyagriv N.	Simhan		MD, MSCR	University of Pittsburgh / Magee-Women's Hospital	Pittsburgh, PA	Site PI	NHLBI nuMoM2b Heart Health Study network
Jill	Tarr		LCSW, ACSW	University of Pittsburgh / Magee-Women's Hospital	Pittsburgh, PA	Lead Coordinator	NHLBI nuMoM2b Heart Health Study network
Julia	Whiteleather		BS	University of Pittsburgh / Magee-Women's Hospital	Pittsburgh, PA	Study Coordinator	NHLBI nuMoM2b Heart Health Study network
Nathan	Blue		MD, MSCI	University of Utah	Salt Lake City, UT	Co-investigator	NHLBI nuMoM2b Heart Health Study network
Kelly	Christensen		RN	University of Utah	Salt Lake City, UT	Study Coordinator	NHLBI nuMoM2b Heart Health Study network
Maggie	Cook		BS	University of Utah	Salt Lake City, UT	Study Coordinator	NHLBI nuMoM2b Heart Health Study network
M. Sean	Esplin		MD	University of Utah	Salt Lake City, UT	Co-investigator	NHLBI nuMoM2b Heart Health Study network
Danielle	Heil		BA	University of Utah	Salt Lake City, UT	Study Coordinator	NHLBI nuMoM2b Heart Health Study network
Torri	Metz		MD	University of Utah	Salt Lake City, UT	Ancillary Study Investigator	NHLBI nuMoM2b Heart Health Study network
Valerie	Morby		RN	University of Utah	Salt Lake City, UT	Study Coordinator	NHLBI nuMoM2b Heart Health Study network
Josh	Oliver		BS	University of Utah	Salt Lake City, UT	Study Coordinator	NHLBI nuMoM2b Heart Health Study network
Dexter	Patterson		BS	University of Utah	Salt Lake City, UT	Study Coordinator	NHLBI nuMoM2b Heart Health Study network
Karen	Schliep		PhD, MSPH	University of Utah	Salt Lake City, UT	Co-investigator	NHLBI nuMoM2b Heart Health Study network
Lauren	Theilen		MD	University of Utah	Salt Lake City, UT	Co-investigator	NHLBI nuMoM2b Heart Health Study network
Sharla	Aldous		RN	University of Utah / Intermountain Medical Center	Murray, UT	Study Coordinator	NHLBI nuMoM2b Heart Health Study network

***First Name and Middle Initial(s)**	***Last Name**	***Suffix (eg, Jr, III)**	Academic Degrees	Institution	Location (city, state/province, country)	Role or Contribution, eg, chair, principal investigator	Group (if more than 1 Group listed in the byline) and/or Subgroup (eg, Steering Committee)
Stacey	Breeze		BA	University of Utah / Intermountain Medical Center	Murray, UT	Study Coordinator	NHLBI nuMoM2b Heart Health Study network
Lindsey	Carlson		PhD	University of Utah / Intermountain Medical Center	Murray, UT	Coordinator	NHLBI nuMoM2b Heart Health Study network
Sandi	Dellerman		RN	University of Utah / Intermountain Medical Center	Murray, UT	Study Coordinator	NHLBI nuMoM2b Heart Health Study network
Holly	Evans		RN	University of Utah / Intermountain Medical Center	Murray, UT	Study Coordinator	NHLBI nuMoM2b Heart Health Study network
Bri	Hobbs		BS	University of Utah / Intermountain Medical Center	Murray, UT	Lead Coordinator	NHLBI nuMoM2b Heart Health Study network
Jessica	Johnson		BSN, RN	University of Utah / Intermountain Medical Center	Murray, UT	Study Coordinator	NHLBI nuMoM2b Heart Health Study network
Jake	Krong		BA	University of Utah / Intermountain Medical Center	Murray, UT	Lead Coordinator	NHLBI nuMoM2b Heart Health Study network
Diana	Magana		BSN, RN	University of Utah / Intermountain Medical Center	Murray, UT	Study Coordinator	NHLBI nuMoM2b Heart Health Study network
Catherine	Meadows		RN	University of Utah / Intermountain Medical Center	Murray, UT	Study Coordinator	NHLBI nuMoM2b Heart Health Study network
Rachel	Moore		BS	University of Utah / Intermountain Medical Center	Murray, UT	Study Coordinator	NHLBI nuMoM2b Heart Health Study network
Valerie	Morby		RN, CCRP	University of Utah / Intermountain Medical Center	Murray, UT	Study Coordinator	NHLBI nuMoM2b Heart Health Study network
Michael	Nunley		MBA-HCM	University of Utah / Intermountain Medical Center	Murray, UT	Lead Coordinator	NHLBI nuMoM2b Heart Health Study network
Kristin	Orton		BSN, RN	University of Utah / Intermountain Medical Center	Murray, UT	Study Coordinator	NHLBI nuMoM2b Heart Health Study network
Midra	Pederson		BSN, RN	University of Utah / Intermountain Medical Center	Murray, UT	Study Coordinator	NHLBI nuMoM2b Heart Health Study network
Sara	St Hilaire		BS	University of Utah / Intermountain Medical Center	Murray, UT	Study Coordinator	NHLBI nuMoM2b Heart Health Study network

***First Name and Middle Initial(s)**	***Last Name**	***Suffix (eg, Jr, III)**	Academic Degrees	Institution	Location (city, state/province, country)	Role or Contribution, eg, chair, principal investigator	Group (if more than 1 Group listed in the byline) and/or Subgroup (eg, Steering Committee)
Kailey	Tingey		RN	University of Utah / Intermountain Medical Center	Murray, UT	Study Coordinator	NHLBI nuMoM2b Heart Health Study network
Nic	Unsworth		BS	University of Utah / Intermountain Medical Center	Murray, UT	Study Coordinator	NHLBI nuMoM2b Heart Health Study network
Bethany	Barone Gibbs		PhD	West Virginia University	Morgantown, WV	Ancillary Study Investigator	NHLBI nuMoM2b Heart Health Study network
