# Supplementary material for: Persistent Short Sleep Duration From Pregnancy to 2 to 7 Years After Delivery and Metabolic Health
Source: JAMA Netw Open. 2024 Dec 26;7(12):e2452204. doi: 10.1001/jamanetworkopen.2024.52204 (PMC11672157; doi:10.1001/jamanetworkopen.2024.52204)
Supplement: Supplement 3. — Data Sharing Statement [file jamanetwopen-e2452204-s003.pdf]

Data Sharing Statement

Kim. Persistent Short Sleep Duration From Pregnancy to 2 to 7 Years After Delivery and Metabolic Health. *JAMA Netw Open*. Published online December 26, 2024. doi:10.1001/jamanetworkopen.2024.52204

Data
Data available: No

Additional Information
Explanation for why data not available: Because of the sensitive nature of the data collected for this study, requests to access the data set from qualified researchers trained in human subject confidentiality protocols will be considered by the data coordinating center. Requests to access the data and materials used for this analysis may be submitted to the nuMoM2b-HHS Study (nulliparous Pregnancy Outcomes Study: Monitoring Mothers-To-be Heart Health Study) at https://numom2b.org.
